# Transcriptome analysis and anaerobic C_4_‐dicarboxylate transport in *Actinobacillus succinogenes*

**DOI:** 10.1002/mbo3.565

**Published:** 2017-12-12

**Authors:** Mi Na Rhie, Byeonghyeok Park, Hyeok‐Jin Ko, In‐Geol Choi, Ok Bin Kim

**Affiliations:** ^1^ Department of Life Science, and Interdisciplinary Program of EcoCreative Ewha Womans University Seoul Korea; ^2^ Department of Biotechnology College of Life Sciences and Biotechnology Korea University Seoul Korea

**Keywords:** *Actinobacillus succinogenes*, C_4_‐dicarboxylate transport, fumarate, transcriptome analysis

## Abstract

A global transcriptome analysis of the natural succinate producer *Actinobacillus succinogenes* revealed that 353 genes were differentially expressed when grown on various carbon and energy sources, which were categorized into six functional groups. We then analyzed the expression pattern of 37 potential C_4_‐dicarboxylate transporters in detail. A total of six transporters were considered potential fumarate transporters: three transporters, Asuc_1999 (Dcu), Asuc_0304 (DASS), and Asuc_0270‐0273 (TRAP), were constitutively expressed, whereas three others, Asuc_1568 (DASS), Asuc_1482 (DASS), and Asuc_0142 (Dcu), were differentially expressed during growth on fumarate. Transport assays under anaerobic conditions with [^14^C]fumarate and [^14^C]succinate were performed to experimentally verify that *A. succinogenes* possesses multiple C_4_‐dicarboxlayte transport systems with different substrate affinities. Upon uptake of 5 mmol/L fumarate, the systems had substrate specificity for fumarate, oxaloacetate, and malate, but not for succinate. Uptake was optimal at pH 7, and was dependent on both proton and sodium gradients. Asuc_1999 was suspected to be a major C_4_‐dicarboxylate transporter because of its noticeably high and constitutive expression. An Asuc_1999 deletion (∆1999) decreased fumarate uptake significantly at approximately 5 mmol/L fumarate, which was complemented by the introduction of Asuc_1999. Asuc_1999 expressed in *Escherichia coli* catalyzed fumarate uptake at a level of 21.6 μmol·gDW ^−1^·min^−1^. These results suggest that C_4_‐dicarboxylate transport in *A. succinogenes* is mediated by multiple transporters, which transport various types and concentrations of C_4_‐dicarboxylates.

## 1 INTRODUCTION

C_4_‐dicarboxylates such as fumarate, succinate, malate, oxaloacetate, and aspartate are relevant intermediates of central metabolism in most living organisms. Because of their direct integration into central metabolic pathways, C_4_‐dicarboxylates serve as good carbon and energy sources for growth. Some bacteria, such as Pseudomonads and Rhizobia, preferentially utilize C_4_‐dicarboxylates over glucose and other sugars (Garcia, Bringhurst, Pinedo, & Gage, 2010; Unden, Strecker, Kleefeld, & Kim, 2016; Valentini & Lapouge, 2013). C_4_‐dicarboxylates are often used as exchange substrates between organisms in symbiotic relationships or in the same ecosystem. In legume‐Rhizobia symbiosis, the bacteroids receive C_4_‐dicarboxylate from plants at the expense of nitrogen fixation, which is achieved by uptake of malate and efflux of aspartate or ammonium (Prell & Poole, 2006; Yurgel & Kahn, 2004). In the bacterial consortium of *Chlorochromatium aggregatum*, the phototrophic epibiont appears to provide α‐ketoglutarate or C_4_‐dicarboxylate for the central motile β‐Proteobacteria in exchange for mobility (Wanner, Vogl, & Overmann, 2008). The genome of the central motile symbiont also contains tripartite ATP‐independent periplasmic (TRAP) dicarboxylate transporters (Liu et al., 2013). The bovine rumen is an ecological niche for many succinate producers such as *Wolinella succinogenes, Actinobacillus succinogenes*,* Mannheimia succiniciproducens*, and *Basfia succiniciproducens* (Baar et al., 2003; Guettler, Rumler, & Jain, 1999; Hong et al., 2004; Kuhnert, Scholten, Haefner, Mayor, & Frey, 2010). Succinate fermenters such as *Prevotella ruminicola*,* Selenomonas ruminantium*, and *Veillonella alcalescens* acquire ATP by decarboxylating succinate to propionate in the rumen (Li et al., 2015). In these contexts, transport systems for C_4_‐dicarboxylates can play important roles in carbon and energy flow between organisms in an ecosystem. Since there are various C_4_‐dicarboxylates and cognate transport systems, the mode of each transport system should meet the functional requirements in its ecological niche. C_4_‐dicarboxylates transporters are classified by the direction of substrate transport into uptake, efflux, and antiport transporters (Janausch, Zientz, Tran, Kröger, & Unden, 2002; Unden et al., 2016).

**Figure 1:**
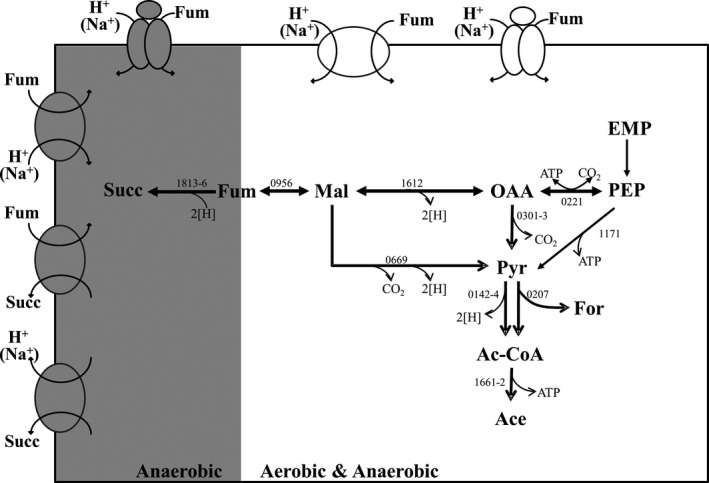
Possible C_4_‐dicarboxylate transport systems related to aerobic and anaerobic metabolism of *A. succinogenes*, based on product analysis from cultivation. The gray background depicts proteins expressed only under anaerobic growth conditions and white background depicts proteins expressed in both aerobic and anaerobic conditions. Suc, succinate; Fum, fumarate; Mal, malate; OAA, oxaloacetate; Pyr, pyruvate; For, formate; Ac‐CoA, acetyl‐CoA; Ace, Acetate; PEP, phosphoenolpyruvate; EMP, Embden‐Meyerhof‐Parnas pathway

*Actinobacillus succinogenes* is a gram‐negative, capnophilic, and facultative aerobic rumen bacterium, and is known as one of the best natural producers of succinate (Guettler et al., 1999; Litsanov, Brocker, Oldiges, & Bott, 2014; McKinlay, Shachar‐Hill, Zeikus, & Vieille, 2007; Rhie et al., 2014). Together with *Mannheimia succiniciproducens* and *Basfia succiniciproducens*,* A. succinogenes* is a nonpathogenic member of the Pasteurellaceae family, and has potential for application in industrial succinate production (Guettler et al., 1999; Kuhnert et al., 2010; Lee, Lee, Hong, & Chang, 2002). The *A. succinogenes* genome possesses several potential C_4_‐dicarboxylate transporters (McKinlay et al., 2010; Rhie et al., 2014), which might be selectively employed under different growth conditions. C_4_‐dicarboxylate consumption and succinate production indicate the presence of various C_4_‐dicarboxylate transporters (Figure 1). *A. succinogenes* grown anaerobically on glucose produces succinate at a stoichiometric ratio of 0.82 succinate/1 glucose (mole/mole) (Rhie et al., 2014), which is evidence for succinate efflux activity (Figure 1). Anaerobic growth on fumarate (or L‐malate) with glycerol resulted in 1.6 succinate/1 fumarate (or 1.2 succinate/1 L‐malate) (Rhie et al., 2014), confirming the existence of C_4_‐dicarboxylate uptake, succinate efflux, and/or C_4_‐dicarboxylate/succinate exchange in *A. succinogenes* (Figure 1). Conversely, aerobic growth on fumarate (or L‐malate) depends entirely on C_4_‐dicarboxylate uptake activity, as only acetate is produced without succinate (Rhie et al., 2014) (Figure 1).

In this study, to survey C_4_‐dicarboxylate transport systems in the transcriptome of *A. succinogenes* grown using different carbon and energy sources, RNA sequencing (RNA‐seq) analysis was performed in aerobic and anaerobic growth conditions. We investigated anaerobic C_4_‐dicarboxylate transport processes involving multiple transporters in *A. succinogenes*. The transporters related to anaerobic fumarate uptake were examined by differentially expressed gene analysis. Among potential C_4_‐dicarboxylate transporters, Asuc_1999 was identified as a main fumarate uptake transporter with constitutive high expression. To validate its cellular function, we experimentally evaluated the in vivo transport activity of Asuc_1999 with a knockout mutant strain and through expression in *Escherichia coli*. This research provides insight into the adaptation of *A. succinogenes* to its ecological niche by utilizing multiple transporter systems to transport different types and concentrations of C_4_‐dicarboxylates.

## 2 MATERIALS AND METHODS

### 2.1 Strains and growth conditions

The strains and plasmids used in this study are shown in Table S1. Subcultures of the *A. succinogenes* strain 130Z were grown in brain–heart infusion (BHI) medium (Difco, USA) at 37°C. Main cultures were grown in modified B‐medium (Guettler et al., 1999) at pH 7.0 containing 8.5 g/L NaH_2_PO_4_·H_2_O (Merck, USA), 15.5 g/L K_2_HPO_4_ (Merck), 10.0 g/L Bacto Tryptone (BD Biosciences, USA), 5.0 g/L Bacto yeast extract (BD Biosciences), and 20 mmol/L NaHCO_3_ (Merck). For growth of the Asuc_1999 mutant strain (LMB018), chloramphenicol (5–15 μg/ml) was added to the medium. *E. coli* strains were grown in Luria‐Bertani (LB) broth at 37°C for subculture and cloning. Main cultures were grown in eM9 medium, which was M9 minimal medium supplemented with acid‐hydrolyzed casein (0.1%, w/v; Neogen, USA) and L‐tryptophan (0.005%, w/v; Deajung, South Korea) (Kim & Unden, 2007). Where necessary, ampicillin (50–100 μg/ml), kanamycin (25–50 μg/ml), spectinomycin (25–50 μg/ml), or chloramphenicol (15–30 μg/ml) was added. D‐Glucose (Samchun, South Korea), disodium fumarate (Sigma, USA), or glycerol (Duksan, South Korea) was added as a carbon and energy source. Bacteria were incubated under anaerobic conditions at 37°C in degassed medium in rubber‐sealed bottles (20 ml medium in 50‐ml bottles) under a stream of N_2_/H_2_ (95:5). Alternatively, bacteria were grown under aerobic conditions by incubation in Erlenmeyer flasks (20 ml medium in 100‐ml flasks) at 37°C with shaking at 180 rpm.

### 2.2 Total mRNA sequencing analysis

Total RNA was isolated from the *A. succinogenes* strain 130Z grown on glucose or fumarate under aerobic or anaerobic condition at midexponential growth phase (OD_600_ of 0.6) using RNAprotect Bacterial Reagent and an RNeasy Mini Kit (Qiagen, Germany), and ribosomal RNA was removed using the Ribo‐Zero rRNA Removal Kit (Epicenter, USA). The mRNA library for next‐generation sequencing (NGS) was prepared using the TruSeq RNA Sample Preparation Kit (Illumina, USA). The mRNA library was sequenced using the Illumina MiSeq platform with MiSeq Reagent Kit v1 (500‐cycles‐PE, Illumina). The sequencing for each growth condition was performed at least in triplicate using three independent culture. Low‐quality (Q < 30) reads were trimmed at the 5′ and 3′ ends using the ShortRead package (Morgan et al., 2009). Bowtie2 (Langmead & Salzberg, 2012) was used for read alignment to the genome sequence of *A. succinogenes* strain 130Z (NCBI RefSeq ID: NC_009655.1). Gene expression profiling and differential gene expression analysis were carried out using the edgeR and DESeq packages in Bioconductor/R (Table S2). Pairwise comparison by condition was performed with four combinations of growth conditions: aerobic growth on glucose versus aerobic growth on fumarate, anaerobic growth on glucose versus anaerobic growth on fumarate with glycerol, aerobic growth on glucose versus anaerobic growth on glucose, and aerobic growth on fumarate versus anaerobic growth on fumarate with glycerol. In this pairwise comparison by condition, genes with |log (base 2) fold change| ≥1 and adjusted *p*‐value≤.1 were designated as differentially expressed (Table S3). Heatmap generation and hierarchical clustering of differentially expressed genes were performed using R with *pheatmap* and *hclust*, respectively. Clustering of differentially expressed genes was performed using *cutree* with *k* = 6.

### 2.3 Molecular genetics methods

#### 2.3.1 Chromosomal gene inactivation of Asuc_1999

Asuc_1999 was amplified by PCR from *A. succinogenes* 130Z chromosomal DNA using the primers Asuc_1999_for (5′‐GTG CTA CGA TGT GCA GAC CG‐3′), and Asuc_1999_SmaI_rev (5′‐GGC CCG GGT CCG ATA TAT TA‐3′). The PCR products were cloned into the multiple cloning site of pGEM^®^‐T Easy (Promega, USA), resulting in the plasmid designated pMB35 (Table S1). The chloramphenicol resistance gene *cat* from pKD3 was inserted into the middle of Asuc_1999 (pMB35) at the SfoI site, resulting in the plasmid designated pMB45. The DNA fragment Asuc_1999::*cat* from pMB45 was transferred into pMB31 (at SphI and PstI), producing the suicide knock‐out vector designated pMB47. The pMB31 plasmid contains a levansucrase gene, *sacB*, from pDM4. The pMB47 plasmid (>2 μg) was transferred into competent *A. succinogenes* cells by electroporation (Micro‐Pulser, Bio‐Rad, USA), and the cells were incubated on BHI agar containing 10 g/L glucose and 10 μg/ml chloramphenicol at 37°C for 3 days. The replacement of genomic Asuc_1999 with Asuc_1999::cat (pMB47) was achieved by double crossover homologous recombination. To eliminate the remaining pMB47, the colonies were transferred twice onto BHI agar containing 100 g/L sucrose and 15 μg/ml chloramphenicol. The Asuc_1999 deletion in *A. succinogenes* (LMB18, Δ1999 strain) was confirmed by PCR and sequencing.

#### 2.3.2 Cloning of Asuc_1999

For expression in *E. coli*, Asuc_1999 was cloned into the pBAD30 vector. The Shine‐Dalgarno sequence (AGGAGG) was introduced by PCR using the primers pBAD30_RBS_for (Eco) (5′‐AGA TAG AGA ATT C**AG GAG G**GA GCT CGG TAC‐3′), pBAD30_(FspI) rev (5′‐CAG TTA ATA GTT **TGC GCA** ACG TTG TTG CCA‐3′), and pBAD30 as template. The PCR product was cloned between the EcoRI and FspI sites of pBAD30, resulting in pMB61 (Table S1). Asuc_1999 was amplified using the primers Asuc_1999_SacI_for (5′‐GAT CTT TG**G AGC TC**G TAT GG‐3′) and Asuc_1999_SphI_rev (5′‐TTC GTT CGT A**GC ATG C**TA TA‐3′). The PCR product was cloned into the MCS site of the vector pMB61, resulting in the plasmid pMB64 (Table S1). For complementation of *A. succinogenes*, the PCR product of Asuc_1999 was cloned into the pLS88 vector, resulting in pMB93.

### 2.4 [^14^C]fumarate/succinate transport assay

Wild‐type *A. succinogenes*, the Δ1999 mutant (LMB18), and LMB18 containing pMB93 were grown anaerobically in 50 mL modified B‐medium with fumarate and glycerol (each 20 mmol/L) at 37°C to an OD_600_ of approximately 0.4. The *E. coli* strain IMW529, containing pMB64, was grown anaerobically on fumarate plus glycerol (each 50 mmol/L) in eM9 medium with L‐arabinose (20 μmol/L) at 37°C to an OD_600_ of approximately 0.7. The harvested cells were washed and resuspended in ice‐cold phosphate buffer (100 mmol/L Na_2_HPO_4_/KH_2_PO_4_ or 100 mmol/L K_2_HPO_4_/KH_2_PO_4_ and 1 mmol/L MgSO_4_, adjusted to pH 7) to an OD_600_ of approximately 7.0, and subsequently degassed on ice. Before commencing the transport assay, the *A. succinogenes* suspension was preincubated at 37°C for 2 min, and the *E. coli* suspension for 5 min with lactose (20 mmol/L). The uptake assay commenced by mixing 50 μl cell suspension with 50 μl of various concentrations of radiolabeled [^14^C]succinate (54.0 mCi/mmol [1,4‐^14^C]succinate; Moravek Biochemicals, USA) or [^14^C]fumarate (55.0 mCi/mmol [2,3‐^14^C]fumarate; Moravek Biochemicals) at 37°C. The reaction was stopped by the addition of 0.9 ml ice‐cold 0.1 mol/L LiCl, followed by rapid vacuum filtration through membrane filters (mixed cellulose ester, diameter 25 mm, 0.2 μm pore size, A020A025A; ADVANTEC^®^, Japan). The filters were washed twice with ice‐cold 0.1 M LiCl, and the radioactivity of the cells was determined using a liquid scintillation counter (Beckman, USA). Transport assays were performed at least in triplicate using three or more independent cell cultures. The transport activities were calculated by measuring the intracellular concentration of [^14^C]succinate or [^14^C]fumarate, based on an OD_600_ of 1.0 corresponding to 313.8 mg dry weight/liter (*A. succinogenes*) and 281 mg dry weight/liter (*E. coli*) (zientz, six, & unden, 1996). To determine the pH‐dependency of transport activity, the initial uptake (1 min) of 5 mmol/L [^14^C]fumarate was determined in cell suspensions prepared in Na^+^/K^+^ phosphate buffer (100 mmol/L Na_2_HPO_4_/KH_2_PO_4_) adjusted to pH values ranging from 4 to 9. The effects of ionophores on fumarate uptake were measured after the initial uptake (1 min) of 5 mmol/L [^14^C]fumarate. The protonophore carbonyl cyanide m‐chlorophenylhydrazone (CCCP, 20 μmol/L; Sigma), and the ionophores monensin (5 μmol/L; Sigma), valinomycin (5 μmol/L; Sigma), and nigericin (2 μmol/L; Sigma) were preincubated with the cell suspensions at 37°C for 2 min before the start of the assay. Competitive inhibition of fumarate uptake was investigated by assaying 4 mmol/L [^14^C]fumarate uptake in the presence of 40 mmol/L unlabeled competitors (fumarate, succinate, oxaloacetate, L‐malate, butyrate, lactate, propionate, pyruvate, acetate, glucose, or citrate) for 1 min.

## 3 RESULTS AND DISCUSSION

### 3.1 Global analysis of differentially expressed genes with various carbon and energy sources

**Figure 2:**
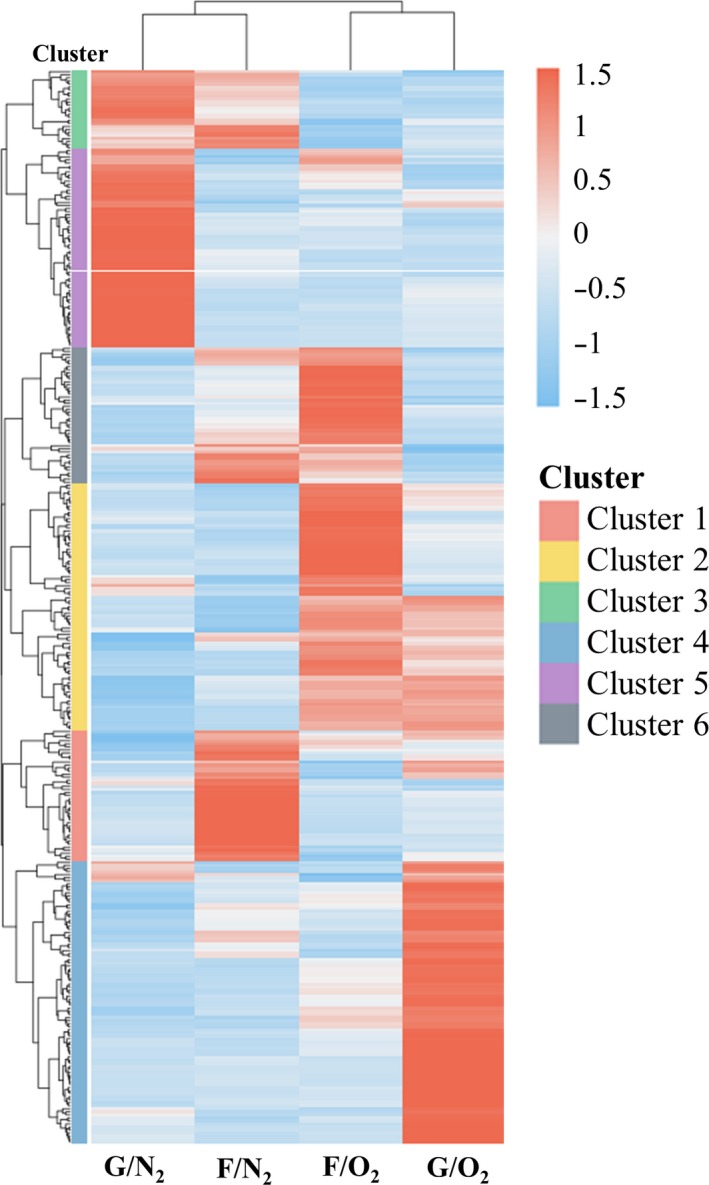
Heatmap of 353 clustered over‐ or underexpressed *A. succinogenes* genes under aerobic or anaerobic growth conditions, with glucose or fumarate. G/N_2_, anaerobic growth on glucose; F/N_2_, anaerobic growth on fumarate with glycerol; F/O_2_, aerobic growth on fumarate; G/O_2_, aerobic growth on glucose

Transcriptional changes in *A. succinogenes* grown with different carbon and energy sources were examined by growing the cells aerobically or anaerobically on either glucose or fumarate (four different conditions; fumarate plus glycerol for anaerobic growth). The full results of expression profiling by high throughput sequencing have been deposited into the GEO database with the accession number GSE92722 (https://www.ncbi.nlm.nih.gov/geo/query/acc.cgi?acc=GSE 92722). Among 2,079 predicted protein‐coding genes in the *A. succinogenes* genome, 353 genes were differentially expressed in at least one pairwise comparison (Figure 2).

**Figure 3:**
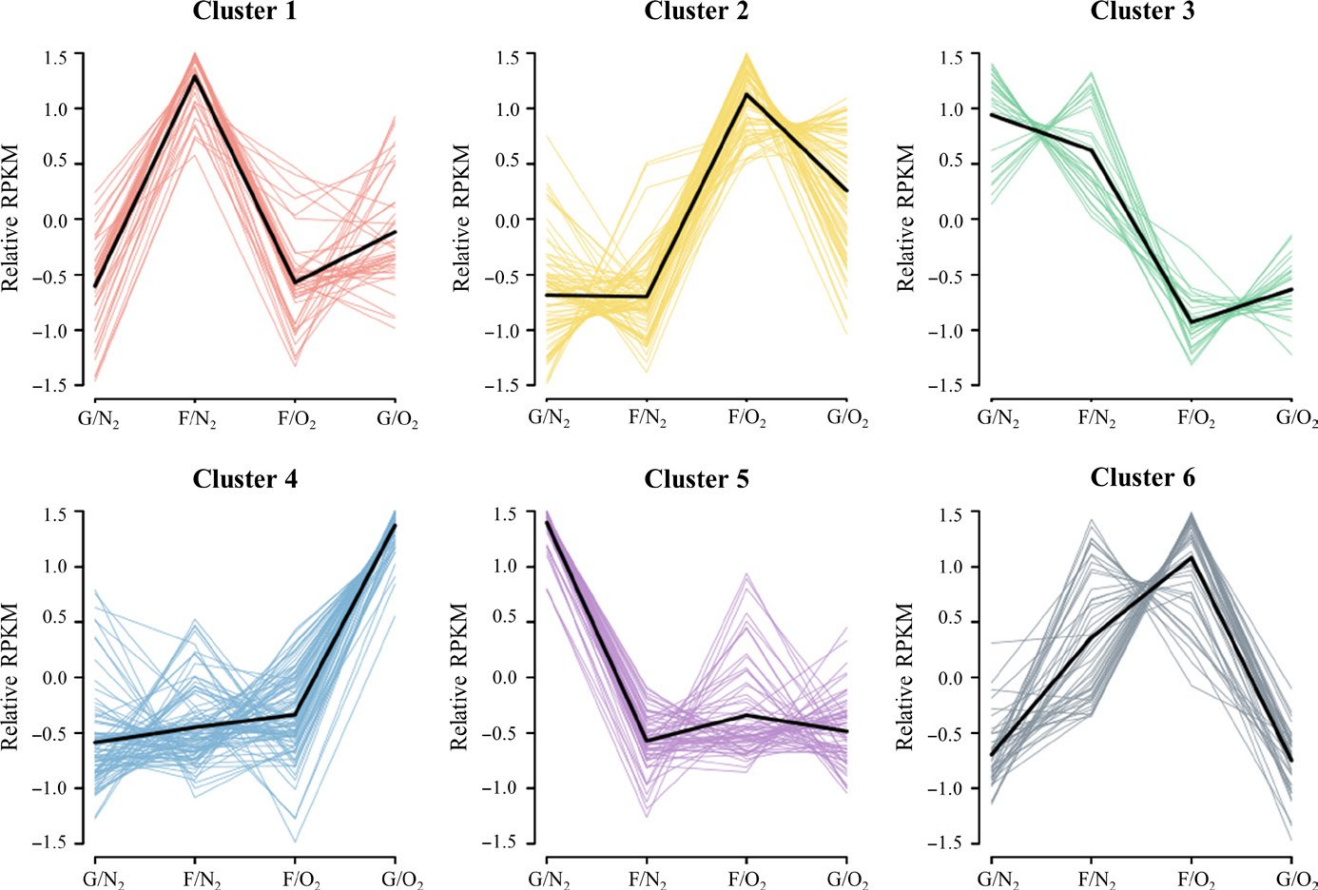
Expression profiles of differentially expressed genes associated with six clusters. Black lines indicate the average profile of each cluster. G/N_2_, anaerobic growth on glucose; F/N_2_, anaerobic growth on fumarate with glycerol; F/O_2_, aerobic growth on fumarate; G/O_2_, aerobic growth on glucose

Next, we classified the 353 differentially expressed genes into six clusters according to their expression patterns (Figure 3, Table S4, Table S5). The Kyoto Encyclopedia of Genes and Genomes (KEGG) orthology database and KEGG pathway annotation were used as references for the cellular functions and associated metabolic pathways of each gene.

Cluster 1 consisted of 43 genes, which had the highest expression levels under anaerobic growth on fumarate with glycerol (Figure 3, Table S4, Table S5). It contained several glycerol‐related genes, including glycerol‐3‐phosphate dehydrogenase (Asuc_0203‐5), glycerophosphoryl diester phosphodiesterase (Asuc_0592), glycerol‐3‐phosphate transporter (Asuc_0593), glycerol uptake facilitator (Asuc_1603), and glycerol kinase (Asuc_1604). A few iron‐related transporter genes (Asuc_1715‐8, Asuc_1014, and Asuc_1820‐1) were also grouped in cluster 1, whereas fumarate related genes were not noted.

Cluster 2 comprised 81 genes that had high expression in both aerobic growth conditions (Figure 3, Table S4, Table S5). Genes involved in aerobic carbon metabolism, including the pyruvate dehydrogenase complex (Asuc_0942‐4), were differentially upregulated in aerobic conditions. In addition, two superoxide dismutases (Asuc_0668 and Asuc_0800) were upregulated, which eliminate reactive oxygen species arising from aerobic respiration.

Cluster 3 was a group of 26 anaerobic‐specific genes (Figure 3, Table S4, Table S5). Interestingly, five genes in the purine metabolism pathway (KEGG accession asu00230) were grouped into cluster 3: ribose‐phosphate pyrophosphokinase (Asuc_1752), phosphor‐ribosylamine‐glycine ligase (Asuc_1148), phosphoribosylglycinamide formyltransferase (Asuc_0730), phosphoribosylformylglycinamidine cyclo‐ligase (Asuc_0729), and phosphor‐ribosylaminoimidazolecarboxamide formyltransferase/inosine monophosphate (IMP) cyclohydrolase (Asuc_1147). These genes are related to the biosynthetic conversion of ribose‐5P to IMP. Since IMP is the precursor of several purine compounds, such as AMP and GMP, this suggests that *A. succinogenes* may require more purine‐containing compounds for anaerobic growth.

Cluster 4 consisted of 93 genes with highest expression under aerobic conditions on glucose (Figure 3, Table S4, Table S5). Four of these genes were members of the biosynthetic module of UDP‐2,3‐diacetamido‐2,3‐dideoxy‐alpha‐D‐glucuronate (Asuc_0108‐0111), and several were aspartate‐related genes: aspartate kinase (Asuc_0925), aspartate transaminase (Asuc_1574), and aspartate‐ammonia ligase (Asuc_0503).

Sixty‐five genes were classified into cluster 5. The expression level of these genes was the highest under anaerobic conditions on glucose (Figure 3, Table S4, Table S5). Genes encoding ribosomal proteins (Asuc_0015, Asuc_0044‐5, Asuc_0520, Asuc_0525, Asuc_0721, Asuc_0774, Asuc_1493‐4, and Asuc_2117) and their accessory proteins were among those classified into this cluster. In addition, members of the beta‐glucoside operon (Asuc_0972‐5) and 11 genes related to the maltose operon (Asuc_0312‐3, Asuc_0315‐0323) were grouped in cluster 5.

Cluster 6 was a fumarate‐specific cluster containing 45 genes (Figure 3, Table S4, Table S5). Genes encoding proteins involved in the ABC transporter system, a ribose transporter (Asuc_0081‐3), an iron (III) transporter (Asuc_1681‐2) and a methylgalactoside transporter (Asuc_1897‐8) were grouped into this cluster.

### 3.2 Differential gene expression of C_4_‐dicarboxylate transport systems with different carbon and energy sources

The genome of *A. succinogenes* revealed 306 transporter genes (Ren, Chen, & Paulsen, 2007), 37 of which potentially encode transport systems for C_4_‐dicarboxylates. Differential expression of potential *A. succinogenes* C_4_‐dicarboxylate transporters was investigated using RNA‐seq analysis during growth on fumarate or glucose under aerobic and anaerobic conditions (Table 1).

**Table 1:** Gene expression related to C_4_‐dicarboxylate transport system based on transcriptome analysis by RNAseq. *A. succinogenes* was grown on glucose or fumarate (fumarate plus glycerol for anaerobic growth) under anaerobic and aerobic conditions

Gene locus	Function	RPKM				Fum O_2_vs. Fum N_2_		Gluc O_2_vs. Gluc N_2_		Fum O_2_vs. Gluc O_2_		Fum N_2_vs. Gluc N_2_	
		FumO_2_	Fum^a^N_2_	GlucO_2_	GlucN_2_	logFC	*p*‐value	logFC	*p*‐value	logFC	*p*‐value	logFC	*p*‐value
The Divalent Anion:Na^+^ Symporter (DASS) Family													
Asuc_0020	anion transporter	87	68	171	91	−0.390	.678	−0.872	.350	0.984	.296	0.491	.600
Asuc_0183	divalent anion:Na^+^ symporter	29	11	39	18	−1.368	.178	−1.025	.286	0.451	.641	0.790	.428
Asuc_0304	Na^+^ dependent C_4_‐dicarboxylate transporter	269	306	266	252	0.176	.849	−0.068	.941	0.030	.974	−0.228	.805
Asuc_1482	divalent anion:Na^+^ symporter	282	78	268	44	−1.914	.047	−2.543	.009	−0.062	.946	−0.702	.454
Asuc_1568	Na^+^ dependent dicarboxylate transporter	1201	223	289	98	−2.433	.012	−1.547	.101	−2.009	.036	−1.138	.224
The C_4_‐Dicarboxylate Uptake (Dcu) Family													
Asuc_0142	anaerobic C_4_‐dicarboxylate transporter DcuB	187	136	202	512	−0.418	.654	1.378	.143	0.062	.947	1.845	.053
Asuc_1999	anaerobic C_4_‐dicarboxylate transporter DcuB	2208	2072	1707	1939	−0.109	.906	0.229	.803	−0.345	.708	−0.022	.981
The C_4_‐Dicarboxylate Uptake C (DcuC) Family													
Asuc_1063	anaerobic C_4_‐dicarboxylate transporter DcuC	**230**	188	97	73	−0.381	.682	−0.366	.694	−1.312	.165	−1.312	.164
The Tripartite ATP‐independent Periplasmic Transporter (TRAP‐T) Family													
Asuc_0146	DctP (SBP)	268	50	81	92	−2.398	.015	0.269	.774	−1.810	.060	0.847	.374
Asuc_0147	DctQ	40	3	15	8	−3.169	.009	−0.645	.566	−1.465	.185	1.086	.370
Asuc_0148	DctM	43	9	24	11	−2.181	.035	−0.968	.324	−0.856	.382	0.352	.727
Asuc_0156	DctM	16	5	9	7	−1.288	.219	−0.279	.786	−0.887	.395	0.115	.912
Asuc_0157	DctQ	2	1	7	3	−0.630	.715	−0.999	.457	1.421	.365	1.189	.438
Asuc_0158	DctP (SBP)	19	5	21	11	−1.572	.145	−0.741	.463	0.027	.979	0.857	.414
Asuc_0270	DctQ	77	87	88	33	0.096	.921	−1.331	.172	0.188	.846	−1.252	.196
Asuc_0271	DctM	80	144	90	51	0.751	.425	−0.818	.384	0.136	.885	−1.447	.127
Asuc_0272	DctP (SBP)	233	203	187	154	−0.211	.821	−0.271	.770	−0.263	.777	−0.337	.717
Asuc_0273	DctP (SBP)	145	181	147	108	0.279	.765	−0.480	.606	0.012	.990	−0.762	.415
Asuc_0366	DctP (SBP)	163	69	44	38	−1.279	.182	−0.170	.858	−1.858	.056	−0.766	.421
Asuc_0367	DctQ	84	25	16	20	−1.695	.092	0.252	.805	−2.305	.027	−0.384	.697
Asuc_0368	DctM	94	44	33	19	−1.103	.249	−0.718	.453	−1.478	.125	−1.109	.248
Asuc_1163	DctP (SBP)	31	18	24	23	−0.865	.398	0.096	.922	−0.484	.628	0.465	.643
Asuc_1164	DctQ	13	5	17	10	−1.037	.392	−0.551	.614	0.281	.809	0.776	.501
Asuc_1165	DctM	37	24	34	24	−0.657	.506	−0.420	.661	−0.202	.835	0.025	.979
Asuc_1577	DctM	49	35	42	54	−0.453	.637	0.458	.627	−0.235	.806	0.667	.482
Asuc_1578	DctQ	43	9	19	30	−1.972	.072	0.625	.539	−1.014	.332	1.584	.138
Asuc_1579	DctP (SBP)	40	27	48	48	−0.664	.500	0.024	.980	0.218	.821	0.900	.355
Asuc_1921	DctM	29	33	36	41	0.182	.852	0.230	.808	0.242	.802	0.276	.773
Asuc_1922	DctQ	6	6	5	5	0.391	.755	−0.405	.719	0.214	.854	−0.595	.605
Asuc_1923	DctP (SBP)	23	32	35	23	0.490	.622	−0.577	.549	0.631	.525	−0.447	.643
Asuc_1956	TAXI (SBP)	23	8	27	12	−1.594	.139	−0.972	.329	0.105	.917	0.725	.492
Asuc_1957	DctM	14	7	18	10	−0.983	.329	−0.794	.415	0.172	.861	0.353	.722
Asuc_1988	TAXI (SBP)	218	173	856	292	−0.370	.692	−1.567	.097	1.941	.043	0.733	.432
Asuc_1989	UspA domain‐containing protein	190	188	789	301	−0.015	.987	−1.342	.153	1.987	.040	0.650	.492
Asuc_1990	DctM	168	129	863	231	−0.460	.619	−1.905	.045	2.326	.016	0.869	.350
Asuc_1991	TAXI (SBP)	683	533	9599	1193	−0.398	.667	−2.920	.003	3.714	.000	1.180	.207
The Tricarboxylate Transporter (TTT) Family													
Asuc_1851	tricarboxylic transport protein	17	20	14	20	0.243	.808	0.464	.630	−0.158	.873	0.051	.958

RPKM, reads per kilobase million; Fum, fumarate; Gluc, glucose; O_2_, aerobic; N_2_, anaerobic; logFC, log_2_ (fold change).

a For anaerobic growth on fumarate, glycerol was supplied as carbon and energy source.

**Figure 4:**
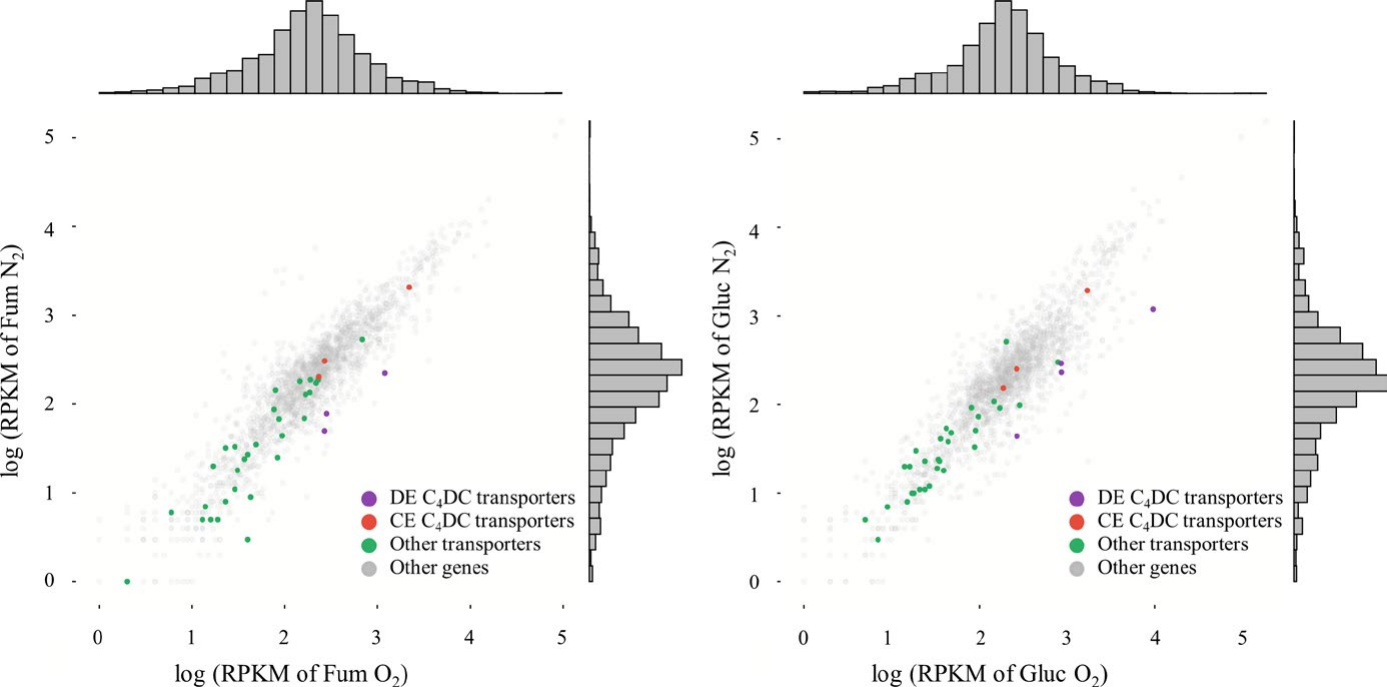
Plot of a log scale of reads per million mapped reads (logRPKM) for 37 potential C_4_‐dicarboxylate transporter genes under aerobic and anaerobic growth conditions with fumarate or glucose. The differentially expressed (DE) C_4_‐dicarboxylate transporters (DE C_4_ DC transporters) and consistently expressed (CE) C_4_‐dicarboxylate transporters (CE C_4_ DC transporters) are indicated in purple and red, respectively. The expression of transporters other than C_4_‐dicarboxylate transporters (Other transporters, green) and the expression of proteins other than transporters (Other genes, gray) are shown for reference

We divided potential C_4_‐dicarboxylate transporter genes into two functional classes based on their gene expression pattern (Figure 4): (1) transporter genes that were differentially expressed (DE) under specific growth conditions were designated as DE C_4_DC transporters. The cut‐off threshold for DE transporter expression was both |log_2_ fold change (logFC)| ≥ 1.0 and *p*‐value ≤.1, and (2) transporter genes that were constitutively expressed (CE) across all experimental conditions were designated as CE C_4_DC transporters. We selected CE transporter genes when their expression was within the top 25% of expression levels in any experimental condition. By this functional categorization, three potential C_4_‐dicarboxylate transporters, Asuc_0272 (TRAP family, clustered with three other subunit genes, Asuc_0270, Asuc_0271, and Asuc_0273), Asuc_0304 (divalent anion‐sodium symporter (DASS) family), and Asuc_1999 (C4‐dicarboxylate uptake (Dcu) family) were designated as CE transporters (Table 1, Figure 4). Among the three, the transcription level of Asuc_1999 was markedly higher than the other transporters in all tested conditions (Table 1), suggesting that it is an important C_4_‐dicarboxylate transporter. The constitutive gene expression of Asuc_0304 has been demonstrated previously by quantitative real‐time PCR (Rhie et al., 2014).

Following aerobic growth on fumarate, Asuc_1568 (DASS family), Asuc_1482 (DASS family), and Asuc_0146 (TRAP family) were classified as DE transporters (Table 1, Figure 4). *A. succinogenes* grown aerobically on fumarate may require only uptake activity for C_4_‐dicarboxylate (Figure 1). In a previous study, Asuc_0304 was shown to be a sodium‐coupled C_4_‐dicarboxylate transporter (ScdA) under aerobic condition, although the gene expression was not affected by the presence of fumarate or oxygen (Rhie et al., 2014).

Anaerobic growth of *A*. *succinogenes* on glucose may require an efflux transporter for succinate (Figure 1), as only Asuc_0142 was differentially expressed under these conditions (Table 1, Figure 4). Conversely, C_4_‐dicarboxylate transporters do not seem to be active during aerobic growth on glucose; the metabolic products of this carbon source were acetate and formate only (Rhie et al., 2014). It is, therefore, interesting that TRAP (Asuc_1988, Asuc_1990, and Asuc_1991) and DASS (Asuc_1482) family transporters were designated as DE transporters under aerobic growth conditions on glucose (Table 1, Figure 4). A TRAP transport system (Asuc_1988–1991) was highly overexpressed during aerobic growth on glucose. The predicted substrates for Asuc_1988–1991 are sugar acids (aldonic or uronic acids), as aldolase (D‐glucose) without a carboxylate moiety did not serve as a substrate for the substrate binding protein of the TRAP transporter (Vetting et al., 2015). Most other TRAP transporters (Asuc_0147–0148, Asuc_0156–0158, Asuc_1163–1165, Asuc_1578, Asuc_1922–1923, and Asuc_1956–1957) and the tripartite tricarboxylate transporter (TTT) family transporter (Asuc_1851) were not differentially expressed under any experimental condition (Table 1).

As a result, the three CE transport systems that may play a basic role in *A. succinogenes* growth in any condition are a TRAP transporter (Asuc_0271‐0273) and a DASS transporter (Asuc_0304), which may be involved in fumarate uptake, and a Dcu transporter (Asuc_1999) which may be involved in fumarate uptake, fumarate/succinate antiport, or succinate efflux. Among the DE transporters, additional DASS transporters (Asuc_1568 and Asuc_1482) may contribute to fumarate uptake during aerobic growth on fumarate, whereas a Dcu transporter (Asuc_0142) may play a role in succinate efflux during anaerobic growth on glucose.

### 3.3 Experimental verification of multiple C_4_‐dicarboxylate transport systems in *A. succinogenes*

**Figure 5:**
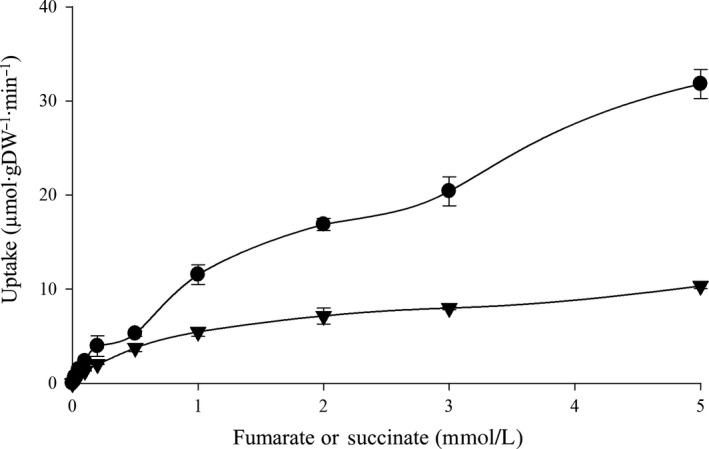
Concentration‐dependent uptake of C_4_‐dicarboxylates in cell suspensions of *A. succinogenes*. The initial uptake (1 min) of [^14^C]fumarate (●) and [^14^C]succinate (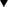) was determined at substrate concentrations from 0 to 5 mmol/L. The assays were performed at least in triplicate using three or more independent cell cultures

The concentration‐dependent uptake of [^14^C]fumarate and [^14^C]succinate in *A. succinogenes* was investigated using filtration assays with cell suspensions of bacteria anaerobically grown on fumarate plus glycerol (Figure 5). The overall uptake activity for fumarate (*V*_max_ 55.8 μmol·gDW^−1^·min^−1^) was 4.7‐fold higher than that of succinate (*V*_max_ 11.9 μmol·gDW^−1^·min^−1^). Interestingly, there were three saturation shoulders for [^14^C]fumarate uptake, in the concentration ranges of (1) 20 μmol/L to 500 μmol/L, (2) 500 μmol/L to 3 mmol/L, and (3) 3 mmol/L to 5 mmol/L, which were supposed to be caused by multiple (at least three) transport systems. The first saturation curve had a *V*_max_ of 7.5 μmol·gDW^−1^·min^−1^ (K_m_ 201.1 μmol/L); the second had a *V*_max_ of 38.0 μmol·gDW^−1^·min^−1^ (K_m_ 2.5 mmol/L); and the third had a *V*_max_ of 58.09 μmol·gDW^−1^·min^−1^ (K_m_ 4.9 mmol/L). Therefore, the anaerobic fumarate uptake of *A. succinogenes* is mediated by multiple transport systems that could be differentiated by substrate affinity. Conversely, anaerobic succinate uptake displayed carrier‐mediated transport with a single saturation point, although it has low affinity with a K_m_ of 1.2 mmol/L (Figure 5).

### 3.4 Properties of anaerobic fumarate uptake in *A. succinogenes*

**Figure 6:**
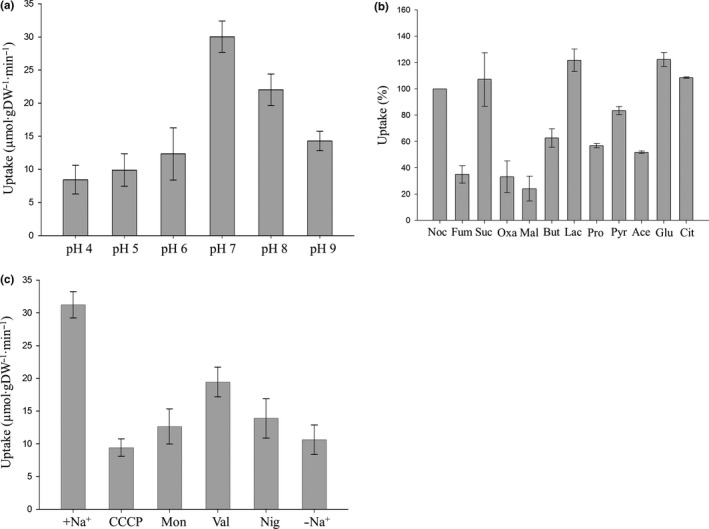
Properties of fumarate uptake in cell suspensions of *A. succinogenes*. (a) Effect of pH. The initial uptake (1 min) of 5 mmol/L [^14^C]fumarate was determined in cells suspended in Na^+^/K^+^ phosphate buffer of pH 4 to 9. (b) Substrate specificity. The initial uptake (1 min) of 4 mmol/L [^14^C]fumarate was determined in the presence of unlabeled competitors (40 mmol/L); 100% uptake activity corresponds to 21.1 μmol·gDW ^−1^·min^−1^. Noc, no competitor; Fum, fumarate; Suc, succinate; OAA, oxaloacetate; Mal, malate; But, butyrate; Lac, lactate; Pro, propionate; Pyr, pyruvate; Ace, acetate; Glc, glucose. (c) Effect of sodium and ionophores. The initial uptake (1 min) of 5 mmol/L [^14^C]fumarate was determined in the presence of ionophores. Cell suspensions were prepared in Na^+^‐containing (Na_2_ HPO_4_/KH_2_ PO_4_) or Na^+^‐free (K_2_ HPO_4_/KH_2_ PO_4_) buffers at pH 7. +Na^+^, Na^+^‐containing buffer; −Na^+^, Na^+^‐free buffer; CCCP, carbonyl cyanide m‐chlorophenylhydrazone; Mon, monensin; Val, valinomycin; Nig, nigericin. The assays were performed at least in triplicate using three or more independent cell cultures

The properties of anaerobic fumarate uptake were studied using 4 (or 5) mmol/L of [^14^C]fumarate to examine the overall uptake activity shown in Figure 5. The pH‐dependency of [^14^C]fumarate uptake was measured in a buffer range from pH 4 to 9 (Figure 6a). The uptake activity was the highest at pH 7 and decreased at acidic or basic pH. This pH profile indicates that dianionic fumarate^2−^ (p*K*a_1_ = 3.03, p*K*a_2_ = 4.44) was preferred by the transporter(s).

The substrate specificity for uptake was investigated by competitive inhibition with a 10‐fold excess of unlabeled C_4_‐dicarboxylates or related substrates (40 mmol/L) to [^14^C]fumarate (4 mmol/L; Figure 6b). The inhibition rates of anaerobic [^14^C]fumarate uptake were 65, 67, and 76% by the unlabeled C_4_‐dicarboxylates fumarate, oxaloacetate, and malate, respectively. However, succinate could not compete with [^14^C]fumarate. The monocarboxylates butyrate (37%), propionate (43%), and acetate (48%) decreased uptake to some extent, but lactate, pyruvate, citrate, and glucose did not inhibit [^14^C]fumarate uptake. The competition assay suggests that fumarate, oxaloacetate, and malate are the preferred substrates for the uptake system(s), with similar specificity. But succinate could not inhibit [^14^C]fumarate uptake.

Various ionophores were used to investigate the driving forces of anaerobic uptake of fumarate (Figure 6c). CCCP is known to collapse the electrochemical proton potential Δp (Nicholls & Ferguson, 2013), and inhibited 70% of the fumarate uptake. The electroneutral H^+^/Na^+^ exchanger monensin inhibited 60% of the fumarate uptake, which was similar to the inhibition observed in Na^+^ free buffer (66%), indicating that fumarate uptake requires a Na^+^‐gradient in addition to proton potential. The electroneutral (nondepolarizing) H^+^/K^+^ exchanger nigericin decreased fumarate uptake by 55%, but the electrical K^+^ uniporter valinomycin only decreased uptake by 38%, meaning that dissipation of the pH gradient negatively affected fumarate uptake. Altogether, these results indicate that the transport system(s) for 5 mmol/L fumarate uptake require(s) electrochemical proton potential Δp, pH gradient ∆pH, and a Na^+^ gradient (ΔΨ_Na+_), whereas a K^+^ gradient (ΔΨ_K+_) appears to be of minor significance.

The assays with *A. succinogenes* showed relatively high background activity, which could be explained by the involvement of more than one transporter in fumarate uptake.

### 3.5 Fumarate transport by the high‐copy transporter Asuc_1999

**Figure 7:**
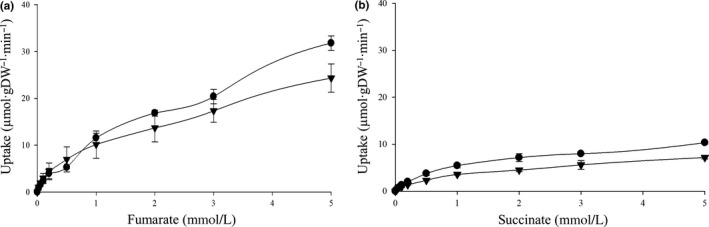
Concentration‐dependent uptake of fumarate in wild type (●) and Δ1999 mutant (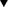) *A. succinogenes* strains. The initial uptake (1 min) of [^14^C]fumarate (a) and [^14^C]succinate (b) was determined at a substrate concentrations from 0 to 5 mmol/L. The assays were performed at least in triplicate using three or more independent cell cultures

Owing to its high transcription under all tested growth conditions, Asuc_1999 was considered a major C_4_‐dicarboxylate transporter (Table 1). Asuc_1999 is 555 amino acids long, and belongs to the Dcu family, showing 85% sequence similarity with *E. coli* DucB (DucB_Ec_, 446 aa), which is the fumarate/succinate antiporter in fumarate respiration (Janausch et al., 2002; Unden et al., 2016). We established a gene knockout system for *A. succinogenes* and constructed the Asuc_1999 deletion (Δ1999) mutant LMB18. In the Δ1999 mutant, the anaerobic fumarate uptake was decreased by 24% at 5 mmol/L fumarate, and the difference between the wild‐type (31.4 μmol·gDW^−1^·min^−1^) and ∆1999 strain (23.9 μmol·gDW^−1^·min^−1^) rates was 7.5 μmol·gDW^−1^·min^−1^ (Figure 7a). The decrease in fumarate uptake in Δ1999 was not detectable at low fumarate concentrations (<1 mmol/L), suggesting that the role of Asuc_1999 could be compensated by other transporters at low concentration. The fumarate uptake of the ∆1999 strain was completely complemented by introduction of Asuc_1999 (pMB93), which was cloned into a broad‐host‐range plasmid of Pasteurellaceae origin, to 31.1 μmol·gDW^−1^·min^−1^ (Table 2).

**Table 2:** Complementation of fumarate uptake of *A. succinogenes* Δ1999 mutant (LMB18) with pMB93 containing Asuc_1999

*A. succinogenes* strains	[^14^C]fumarate uptake (μmol·gDW^−1^·min^−1^)
wild type	31.4 ± 2.1
LMB18	23.9 ± 1.5
LMB18 + pMB93	31.1 ± 3.3

The initial uptake (1 min) was determined with 5 mmol/L [^14^C]fumarate. The assays were performed at least in triplicate using three or more independent cell cultures.

**Figure 8:**
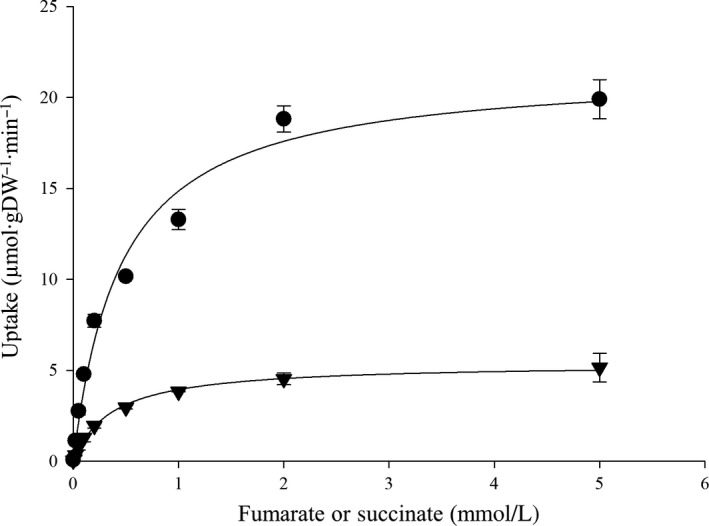
Anaerobic C_4_‐dicarboxylate uptake of Asuc_1999 in the *E. coli* strain IMW529. The initial uptake (1 min) of [^14^C]fumarate (●) or [^14^C]succinate (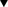) was determined at a substrate concentrations from 0 to 5 mmol/L. The assays were performed at least in triplicate using three or more independent cell cultures

In addition, the anaerobic uptake by Asuc_1999 was determined directly, albeit heterologously, by cloning Asuc_1999 into a low‐copy expression plasmid (pMB64) and expressing it in the *E. coli* strain IMW529 (Figure 8), which is deficient in anaerobic fumarate transport (Kim & Unden, 2007). The C_4_‐dicarboxylate uptake activity of pMB64 showed a clear carrier‐mediated fashion dependent on substrate concentration. The heterologous fumarate uptake activity revealed a *V*_max_ of 21.6 μmol·gDW^−1^·min^−1^ with *K*_m_ of 452 μmol/L. The uptake activity for succinate (*V*_max_ 5.4 μmol∙gDW^−1^∙min^−1^; *K*_m_ 364 μmol/L) was fourfold lower than fumarate (Figure 8), corresponding that Asuc_1999 knockout only slightly decreased succinate uptake (Figure 7b).

## 4 CONCLUSION

*A. succinogenes* grows well on fumarate plus glycerol under anaerobic conditions. Supplied fumarate is completely converted into succinate, and half of the supplied glycerol is also converted to succinate. As a result, it is assumed that growth requires transporters for fumarate uptake and succinate efflux. Anaerobic transport assays revealed that multiple transport systems in *A. succinogenes* catalyzing fumarate uptake, with distinct substrate affinity and activity. RNAseq analysis showed that three potential C_4_‐dicarboxylate transport systems, namely Asuc_0271‐0273 (TRAP), Asuc_0304 (DASS), and Asuc_1999 (Dcu), were expressed during anaerobic growth on fumarate plus glycerol. The transcription level of Asuc_1999 was markedly higher than that of other C_4_‐dicarboxylate transport genes under all tested growth conditions (aerobic and anaerobic conditions with glucose or fumarate). The deletion of Asuc_1999 caused a significant decrease in fumarate uptake at high fumarate concentrations, which was complemented by reintroducing Asuc_1999. In addition, Asuc_1999 heterologously expressed in *E. coli* catalyzed fumarate uptake. Overall, the results indicate that Asuc_1999 could be a common C_4_‐dicarboxylate transporter exhibiting high fumarate uptake activity.

## CONFLICTS OF INTEREST

The authors declare that they have no conflicts of interest.
