# Supplementary figures and images for: Transcriptome analysis and anaerobic C4‐dicarboxylate transport in Actinobacillus succinogenes
Source: Microbiologyopen. 2017 Dec 12;7(3):e00565. doi: 10.1002/mbo3.565 (PMC6011838; doi:10.1002/mbo3.565)

FIG. S1 A

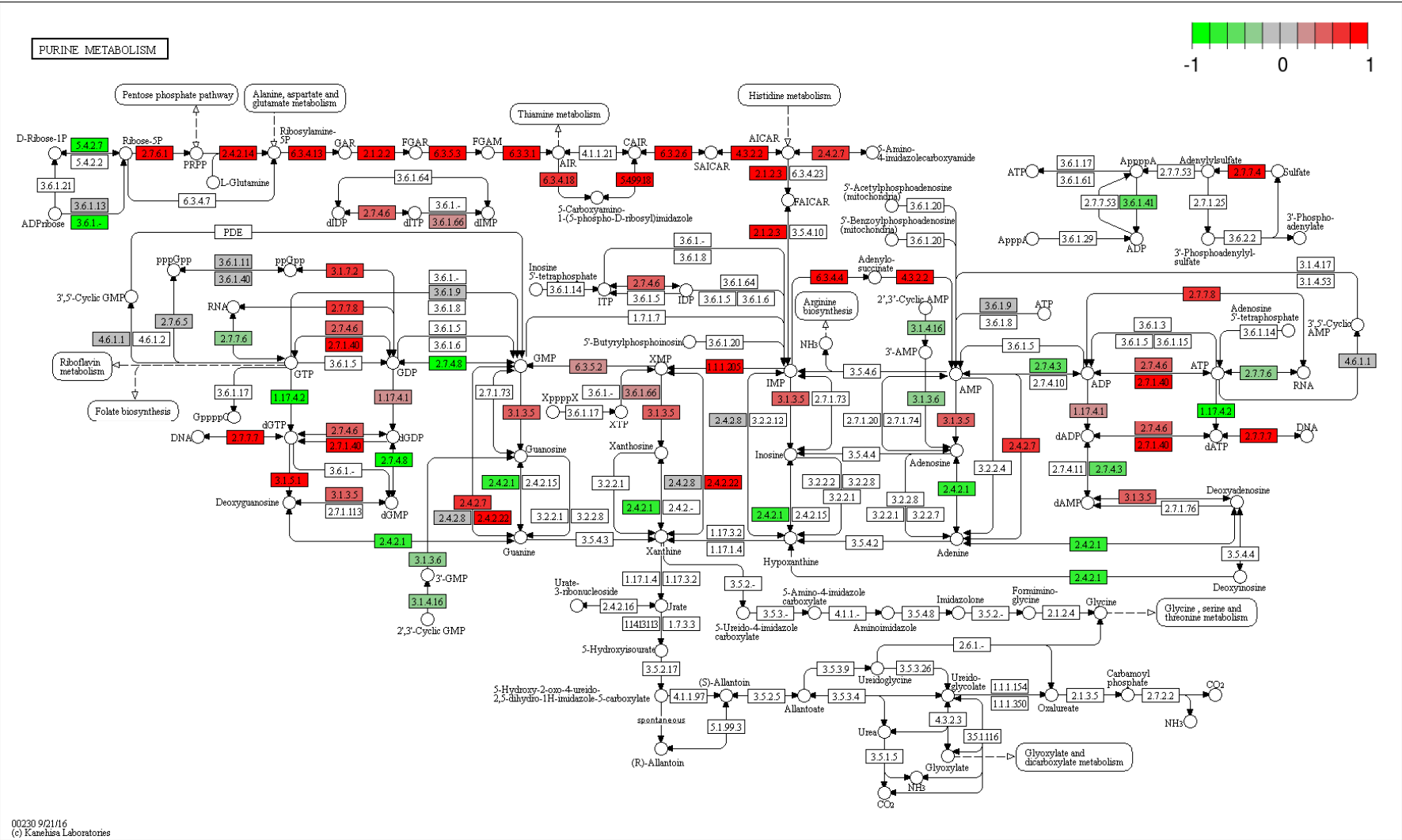

Supplement: Supplementary file 1 [file MBO3-7-e00565-s001.pdf]

FIG. S1 B

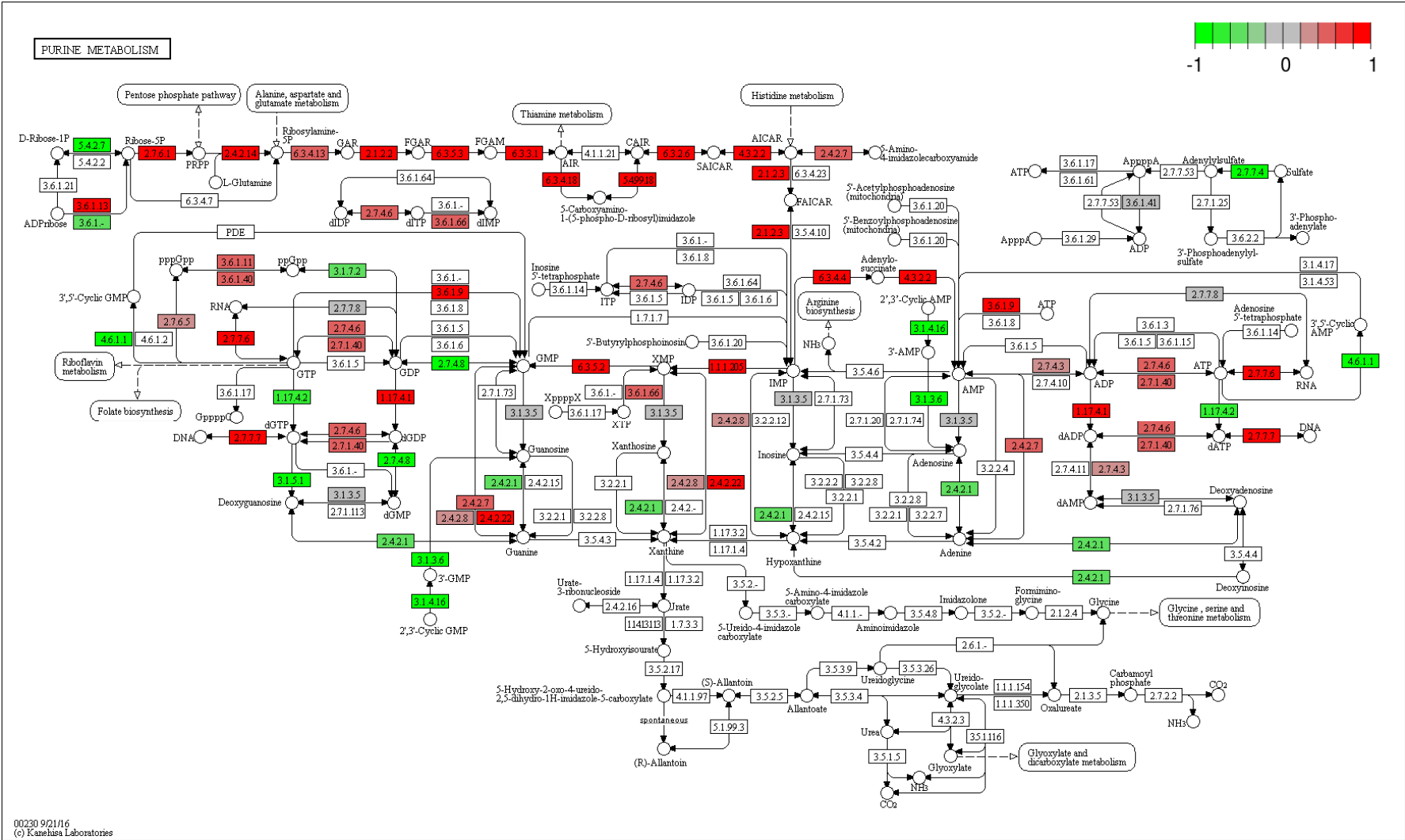

Supplement: Supplementary file 2 [file MBO3-7-e00565-s002.pdf]

FIG. S2 A

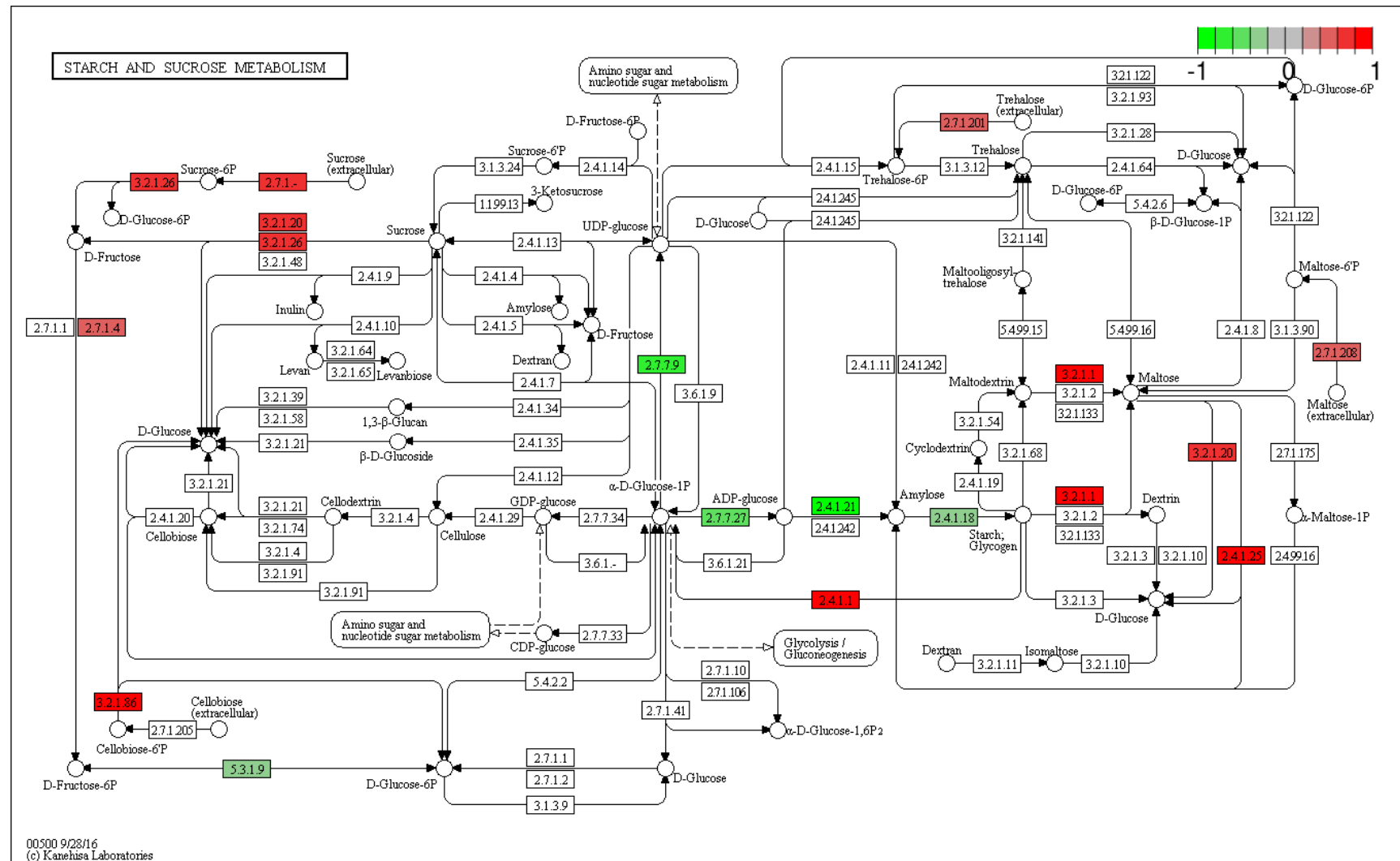

Supplement: Supplementary file 3 [file MBO3-7-e00565-s003.pdf]

FIG. S2 B

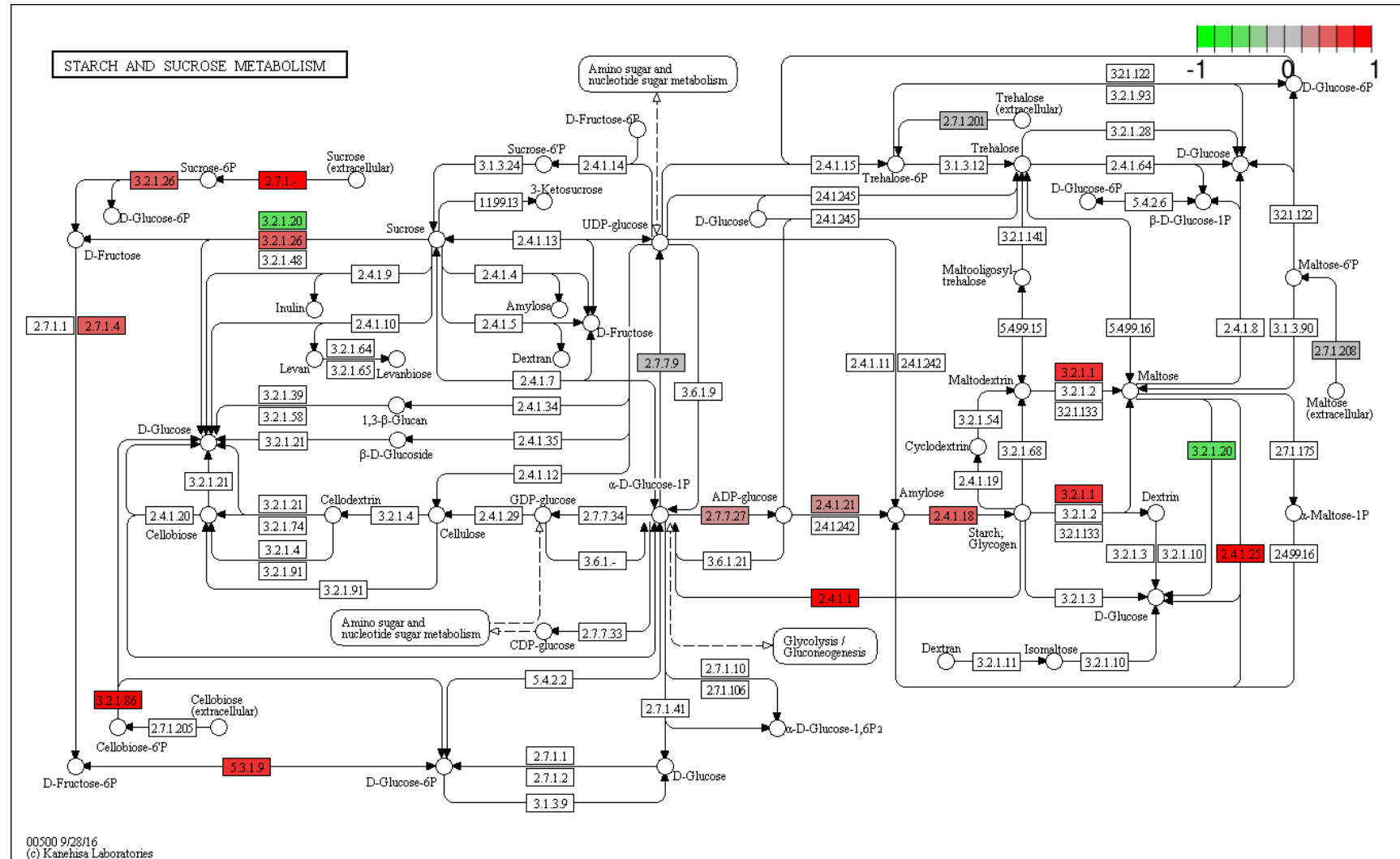

Supplement: Supplementary file 4 [file MBO3-7-e00565-s004.pdf]
