# Supplementary material for: Transcriptome analysis and anaerobic C4‐dicarboxylate transport in Actinobacillus succinogenes
Source: Microbiologyopen. 2017 Dec 12;7(3):e00565. doi: 10.1002/mbo3.565 (PMC6011838; doi:10.1002/mbo3.565)
Supplement: Supplementary file 5 [file MBO3-7-e00565-s005.docx]

**Table S1.** Strains and plasmids used in this study

| Strains or Plasmids | Relevant genotype | Source /References |
| --- | --- | --- |
| **Strains** |  |  |
| *Actinobacillus succinogenes* 130Z | Wild-type strain (DSM 22257) | DSMZ |
| LMB18 | *A. succinogenes* 130Z*,* but Δ*Asuc_1999*::*cat* | This study |
|  |  |  |
| *Escherichia coli* DH5α | *fhuA2 lac(del)U169 phoA glnV44 Φ80' lacZ(del)M15 gyrA96 recA1 relA1 endA1 thi-1 hsdR17* | Taylor *et al.* (1993) |
| IMW529 | LJ1, but *dcuA::spc^R^, dcuB::kan^R^, dcuC::Tn10 (cm^R^), citT::kan^R^*, *ΔttdT* | Kim and Unden (2007) |
|  |  |  |
| **Plasmids** |  |  |
| pUC19 | Cloning vector, amp^r^ | Enzynomics Corporation |
| pDM4 | oriR6K, Cloning vector, suicide vector, *sacB*, cam^r^ | Mou *et al*. (2013) |
| pGEM^®^-T Easy | Cloning vector, amp^r^ | Promega Corporation |
| pKD3 | oriR, *cat bla* Δ*(phoB-phoR)580 galU95* Δ*uidA3::pir* Δ*ndA::*FRT | Datsenko and Wanner (2000) |
| pBAD30 | *araC, araBAD* promoter, pACYC184, *bla,* amp^r^ | Guzman *et al.* (1995) |
| pLS88 | Shuttle vector (ATCC 86980), kan^r^, str^r^, sul^r^ | ATCC |
| pMB31 | pUC19 with complete gene *sacB* (1422 bp) form pDM4, amp^r^ | This study |
| pMB35 | pGEM^®^-T Easy with complete gene *Asuc*_*1999* (1668 bp) of *A.succinogenes*, amp^r^ | This study |
| pMB45 | pMB35 with *Asuc_1999*::*cat*, amp^r^ | This study |
| pMB47 | pMB31 with *Asuc_1999*::*cat* and *sacB*, amp^r^ | This study |
| pMB61 | pBAD30 with Shine-Dalgarno (SD) sequence, amp^r^ | This study |
| pMB64 | pMB61 with *Asuc_1999*, amp^r^ | This study |
| pMB93 | pLS88 with *Asuc_1999*, kan^r^, str^r^ | This study |
