# Supplementary material for: Transcriptome analysis and anaerobic C4‐dicarboxylate transport in Actinobacillus succinogenes
Source: Microbiologyopen. 2017 Dec 12;7(3):e00565. doi: 10.1002/mbo3.565 (PMC6011838; doi:10.1002/mbo3.565)
Supplement: Supplementary file 6 [file MBO3-7-e00565-s006.docx]

**Table S2.** Statistics of reads used in the mRNA sequencing of this study

| Growth condition | Anaerobic  glucose | | | Aerobic  glucose | | | Anaerobic  fumarate + glycerol | | | Aerobic  fumarate | | | |
| --- | --- | --- | --- | --- | --- | --- | --- | --- | --- | --- | --- | --- | --- |
| Replication Number | 1 | 2 | 3 | 1 | 2 | 3 | 1 | 2 | 3 | 1 | 2 | 3 |  |
| Number of paired-end raw reads | 1247364 | 1167378 | 1693132 | 1356194 | 340889 | 459718 | 1113151 | 1167429 | 1165472 | 204034 | 371626 | 613271 |  |
| Number of aligned reads (R1) | 1143989 | 1134926 | 1611772 | 1322138 | 330783 | 237309 | 1071798 | 1159362 | 1133393 | 197792 | 367355 | 606297 |  |
| Number of aligned reads (R2) | 1138727 | 1131035 | 1603662 | 1318076 | 329480 | 231607 | 1067650 | 1156243 | 1129065 | 197138 | 366189 | 603909 |  |
| Alignment rate of reads (R1, %) | 91.7 | 97.2 | 95.2 | 97.5 | 97.0 | 51.6 | 96.3 | 99.3 | 97.2 | 96.9 | 98.9 | 98.9 |  |
| Alignment rate of reads (R2, %) | 91.3 | 96.9 | 94.7 | 97.2 | 96.7 | 50.4 | 95.9 | 99.0 | 96.9 | 96.6 | 98.5 | 98.5 |  |
