# Supplementary material for: Transcriptome analysis and anaerobic C4‐dicarboxylate transport in Actinobacillus succinogenes
Source: Microbiologyopen. 2017 Dec 12;7(3):e00565. doi: 10.1002/mbo3.565 (PMC6011838; doi:10.1002/mbo3.565)
Supplement: Supplementary file 7 [file MBO3-7-e00565-s007.docx]

**Table S3.** Significantly induced or repressed KEGG pathways in condition-by-condition pairwise enrichment analysis.

| KEGG pathway number | Induced condition | P-value | Number of genes in pathway |
| --- | --- | --- | --- |
| **Fumarate anaerobic vs. Fumarate aerobic** | | | |
| asu00230 Purine metabolism | Fumarate anaerobic | 0.0019 | 56 |
| asu03010 Ribosome | Fumarate anaerobic | 0.0030 | 54 |
| asu01110 Biosynthesis of secondary metabolites | Fumarate anaerobic | 0.0113 | 203 |
| asu00564 Glycerophospholipid metabolism | Fumarate anaerobic | 0.0168 | 19 |
| asu03060 Protein export | Fumarate anaerobic | 0.0187 | 17 |
| asu00970 Aminoacyl-tRNA biosynthesis | Fumarate anaerobic | 0.0280 | 24 |
| asu01100 Metabolic pathways | Fumarate anaerobic | 0.0331 | 450 |
| asu03070 Bacterial secretion system | Fumarate anaerobic | 0.0372 | 14 |
| asu03440 Homologous recombination | Fumarate anaerobic | 0.0518 | 26 |
| asu03018 RNA degradation | Fumarate anaerobic | 0.0585 | 14 |
| asu00670 One carbon pool by folate | Fumarate anaerobic | 0.0747 | 10 |
| asu00561 Glycerolipid metabolism | Fumarate anaerobic | 0.0771 | 12 |
| asu00053 Ascorbate and aldarate metabolism | Fumarate aerobic | 0.0089 | 14 |
| asu00040 Pentose and glucuronate interconversions | Fumarate aerobic | 0.0770 | 20 |
| asu00270 Cysteine and methionine metabolism | Fumarate aerobic | 0.0911 | 17 |
| **Glucose anaerobic vs. Glucose aerobic** | | | |
| asu03010 Ribosome | Glucose anaerobic | 1.11E-11 | 54 |
| asu03060 Protein export | Glucose anaerobic | 0.0034 | 17 |
| asu03070 Bacterial secretion system | Glucose anaerobic | 0.0060 | 14 |
| asu00230 Purine metabolism | Glucose anaerobic | 0.0062 | 56 |
| asu00970 Aminoacyl-tRNA biosynthesis | Glucose anaerobic | 0.0175 | 24 |
| asu00061 Fatty acid biosynthesis | Glucose anaerobic | 0.0261 | 14 |
| asu01212 Fatty acid metabolism | Glucose anaerobic | 0.0261 | 14 |
| asu00052 Galactose metabolism | Glucose anaerobic | 0.0306 | 10 |
| asu01110 Biosynthesis of secondary metabolites | Glucose anaerobic | 0.0405 | 203 |
| asu02010 ABC transporters | Glucose anaerobic | 0.0458 | 104 |
| asu01100 Metabolic pathways | Glucose anaerobic | 0.0500 | 450 |
| asu00900 Terpenoid backbone biosynthesis | Glucose anaerobic | 0.0602 | 10 |
| asu00670 One carbon pool by folate | Glucose anaerobic | 0.0621 | 10 |
| asu00920 Sulfur metabolism | Glucose aerobic | 0.0027 | 17 |
| asu00053 Ascorbate and aldarate metabolism | Glucose aerobic | 0.0618 | 14 |
| asu00040 Pentose and glucuronate interconversions | Glucose aerobic | 0.0656 | 20 |
| asu00760 Nicotinate and nicotinamide metabolism | Glucose aerobic | 0.0729 | 11 |
| **Glucose anaerobic vs. Fumarate anaerobic** | | | |
| asu03010 Ribosome | Glucose anaerobic | 0.0005 | 54 |
| asu00500 Starch and sucrose metabolism | Glucose anaerobic | 0.0479 | 17 |
| asu00052 Galactose metabolism | Glucose anaerobic | 0.0689 | 10 |
| asu01230 Biosynthesis of amino acids | Glucose anaerobic | 0.0994 | 100 |
| asu00564 Glycerophospholipid metabolism | Fumarate anaerobic | 0.0261 | 19 |
| asu00920 Sulfur metabolism | Fumarate anaerobic | 0.0436 | 17 |
| asu00561 Glycerolipid metabolism | Fumarate anaerobic | 0.0785 | 12 |
| asu00290 Valine, leucine and isoleucine biosynthesis | Fumarate anaerobic | 0.0994 | 13 |
| **Glucose aerobic vs. Fumarate aerobic** | | | |
| asu00500 Starch and sucrose metabolism | Glucose aerobic | 0.0259 | 17 |
| asu00920 Sulfur metabolism | Glucose aerobic | 0.0399 | 17 |
| asu02060 Phosphotransferase system (PTS) | Glucose aerobic | 0.0432 | 24 |
| asu03018 RNA degradation | Glucose aerobic | 0.0564 | 14 |
| asu00051 Fructose and mannose metabolism | Glucose aerobic | 0.0641 | 31 |
| asu00260 Glycine, serine and threonine metabolism | Glucose aerobic | 0.0657 | 22 |
| asu00300 Lysine biosynthesis | Glucose aerobic | 0.0997 | 13 |
| asu00564 Glycerophospholipid metabolism | Glucose aerobic | 0.0999 | 19 |
| asu03010 Ribosome | Fumarate aerobic | 0.0087 | 54 |
| asu02010 ABC transporters | Fumarate aerobic | 0.0440 | 104 |
