# Supplementary material for: Transcriptome analysis and anaerobic C4‐dicarboxylate transport in Actinobacillus succinogenes
Source: Microbiologyopen. 2017 Dec 12;7(3):e00565. doi: 10.1002/mbo3.565 (PMC6011838; doi:10.1002/mbo3.565)
Supplement: Supplementary file 8 [file MBO3-7-e00565-s008.docx]

**Table S4.** List of hierarchically clustered 353 differentially expressed genes and their KEGG annotations. KEGG orthology (KO) annotations were downloaded from KEGG API (<http://rest.kegg.jp>/list /asu).

| cluster | gene | KO annotation |
| --- | --- | --- |
| cluster 1 | Asuc_0202 | K07447 putative holliday junction resolvase [EC:3.1.-.-] |
| cluster 1 | Asuc_0203 | K00113 glycerol-3-phosphate dehydrogenase subunit C [EC:1.1.5.3] |
| cluster 1 | Asuc_0204 | K00112 glycerol-3-phosphate dehydrogenase subunit B [EC:1.1.5.3] |
| cluster 1 | Asuc_0205 | K00111 glycerol-3-phosphate dehydrogenase [EC:1.1.5.3] |
| cluster 1 | Asuc_0213 | no KO assigned |
| cluster 1 | Asuc_0243 | no KO assigned |
| cluster 1 | Asuc_0244 | K09893 regulator of ribonuclease activity B |
| cluster 1 | Asuc_0269 | no KO assigned |
| cluster 1 | Asuc_0288 | K02796 PTS system, mannose-specific IID component |
| cluster 1 | Asuc_0350 | K03667 ATP-dependent HslUV protease ATP-binding subunit HslU |
| cluster 1 | Asuc_0592 | K01126 glycerophosphoryl diester phosphodiesterase [EC:3.1.4.46] |
| cluster 1 | Asuc_0593 | K02445 MFS transporter, OPA family, glycerol-3-phosphate transporter |
| cluster 1 | Asuc_0706 | K04014 protein NrfC |
| cluster 1 | Asuc_0707 | K04015 protein NrfD |
| cluster 1 | Asuc_0786 | K02065 phospholipid/cholesterol/gamma-HCH transport system ATP-binding protein |
| cluster 1 | Asuc_0912 | K03568 TldD protein |
| cluster 1 | Asuc_1014 | K04759 ferrous iron transport protein B |
| cluster 1 | Asuc_1015 | no KO assigned |
| cluster 1 | Asuc_1023 | K03655 ATP-dependent DNA helicase RecG [EC:3.6.4.12] |
| cluster 1 | Asuc_1068 | no KO assigned |
| cluster 1 | Asuc_1172 | no KO assigned |
| cluster 1 | Asuc_1241 | no KO assigned |
| cluster 1 | Asuc_1290 | no KO assigned |
| cluster 1 | Asuc_1334 | no KO assigned |
| cluster 1 | Asuc_1420 | no KO assigned |
| cluster 1 | Asuc_1439 | K05881 PTS hybrid protein |
| cluster 1 | Asuc_1440 | K05879 dihydroxyacetone kinase, C-terminal domain [EC:2.7.1.-] |
| cluster 1 | Asuc_1555 | K08309 soluble lytic murein transglycosylase [EC:3.2.1.-] |
| cluster 1 | Asuc_1557 | K03814 monofunctional biosynthetic peptidoglycan transglycosylase [EC:2.4.1.-] |
| cluster 1 | Asuc_1603 | K02440 glycerol uptake facilitator protein |
| cluster 1 | Asuc_1604 | K00864 glycerol kinase [EC:2.7.1.30] |
| cluster 1 | Asuc_1686 | K00381 sulfite reductase (NADPH) hemoprotein beta-component [EC:1.8.1.2] |
| cluster 1 | Asuc_1687 | K00380 sulfite reductase (NADPH) flavoprotein alpha-component [EC:1.8.1.2] |
| cluster 1 | Asuc_1690 | K00390 phosphoadenosine phosphosulfate reductase [EC:1.8.4.8 1.8.4.10] |
| cluster 1 | Asuc_1715 | K02010 iron(III) transport system ATP-binding protein [EC:3.6.3.30] |
| cluster 1 | Asuc_1716 | K02011 iron(III) transport system permease protein |
| cluster 1 | Asuc_1717 | K02010 iron(III) transport system ATP-binding protein [EC:3.6.3.30] |
| cluster 1 | Asuc_1718 | K02012 iron(III) transport system substrate-binding protein |
| cluster 1 | Asuc_1820 | K02016 iron complex transport system substrate-binding protein |
| cluster 1 | Asuc_1821 | K02013 iron complex transport system ATP-binding protein [EC:3.6.3.34] |
| cluster 1 | Asuc_1843 | no KO assigned |
| cluster 1 | Asuc_1949 | K02028 polar amino acid transport system ATP-binding protein [EC:3.6.3.21] |
| cluster 1 | Asuc_2054 | K00428 cytochrome c peroxidase [EC:1.11.1.5] |
| cluster 2 | Asuc_0058 | no KO assigned |
| cluster 2 | Asuc_0059 | no KO assigned |
| cluster 2 | Asuc_0060 | no KO assigned |
| cluster 2 | Asuc_0072 | K06889 uncharacterized protein |
| cluster 2 | Asuc_0073 | K07478 putative ATPase |
| cluster 2 | Asuc_0074 | K03304 tellurite resistance protein |
| cluster 2 | Asuc_0075 | no KO assigned |
| cluster 2 | Asuc_0123 | K08319 putative dehydrogenase [EC:1.1.-.-] |
| cluster 2 | Asuc_0143 | no KO assigned |
| cluster 2 | Asuc_0145 | K01812 glucuronate isomerase [EC:5.3.1.12] |
| cluster 2 | Asuc_0146 | no KO assigned |
| cluster 2 | Asuc_0147 | no KO assigned |
| cluster 2 | Asuc_0148 | no KO assigned |
| cluster 2 | Asuc_0159 | no KO assigned |
| cluster 2 | Asuc_0173 | K17204 erythritol transport system ATP-binding protein |
| cluster 2 | Asuc_0233 | no KO assigned |
| cluster 2 | Asuc_0268 | K08092 3-dehydro-L-gulonate 2-dehydrogenase [EC:1.1.1.130] |
| cluster 2 | Asuc_0351 | K11933 NADH oxidoreductase Hcr [EC:1.-.-.-] |
| cluster 2 | Asuc_0352 | K05601 hydroxylamine reductase [EC:1.7.99.1] |
| cluster 2 | Asuc_0395 | no KO assigned |
| cluster 2 | Asuc_0396 | K07126 uncharacterized protein |
| cluster 2 | Asuc_0403 | K13954 alcohol dehydrogenase [EC:1.1.1.1] |
| cluster 2 | Asuc_0447 | K02526 2-keto-3-deoxygluconate permease |
| cluster 2 | Asuc_0449 | K03089 RNA polymerase sigma-32 factor |
| cluster 2 | Asuc_0494 | K01804 L-arabinose isomerase [EC:5.3.1.4] |
| cluster 2 | Asuc_0495 | K02529 LacI family transcriptional regulator |
| cluster 2 | Asuc_0499 | K10543 D-xylose transport system substrate-binding protein |
| cluster 2 | Asuc_0505 | K07391 magnesium chelatase family protein |
| cluster 2 | Asuc_0507 | no KO assigned |
| cluster 2 | Asuc_0553 | no KO assigned |
| cluster 2 | Asuc_0558 | K05809 ribosome-associated inhibitor A |
| cluster 2 | Asuc_0668 | K04564 superoxide dismutase, Fe-Mn family [EC:1.15.1.1] |
| cluster 2 | Asuc_0800 | K04565 superoxide dismutase, Cu-Zn family [EC:1.15.1.1] |
| cluster 2 | Asuc_0864 | K04487 cysteine desulfurase [EC:2.8.1.7] |
| cluster 2 | Asuc_0865 | K04488 nitrogen fixation protein NifU and related proteins |
| cluster 2 | Asuc_0866 | K13628 iron-sulfur cluster assembly protein |
| cluster 2 | Asuc_0889 | K03299 gluconate:H+ symporter, GntP family |
| cluster 2 | Asuc_0931 | K00147 glutamate-5-semialdehyde dehydrogenase [EC:1.2.1.41] |
| cluster 2 | Asuc_0942 | K00163 pyruvate dehydrogenase E1 component [EC:1.2.4.1] |
| cluster 2 | Asuc_0943 | K00627 pyruvate dehydrogenase E2 component (dihydrolipoamide acetyltransferase) [EC:2.3.1.12] |
| cluster 2 | Asuc_0944 | K00382 dihydrolipoamide dehydrogenase [EC:1.8.1.4] |
| cluster 2 | Asuc_1157 | K03299 gluconate:H+ symporter, GntP family |
| cluster 2 | Asuc_1192 | K07275 outer membrane protein |
| cluster 2 | Asuc_1213 | no KO assigned |
| cluster 2 | Asuc_1214 | K07334 proteic killer suppression protein |
| cluster 2 | Asuc_1218 | no KO assigned |
| cluster 2 | Asuc_1227 | no KO assigned |
| cluster 2 | Asuc_1248 | no KO assigned |
| cluster 2 | Asuc_1307 | no KO assigned |
| cluster 2 | Asuc_1322 | K06999 phospholipase/carboxylesterase |
| cluster 2 | Asuc_1326 | no KO assigned |
| cluster 2 | Asuc_1327 | no KO assigned |
| cluster 2 | Asuc_1328 | K03675 glutaredoxin 2 |
| cluster 2 | Asuc_1351 | K00705 4-alpha-glucanotransferase [EC:2.4.1.25] |
| cluster 2 | Asuc_1366 | K06910 uncharacterized protein |
| cluster 2 | Asuc_1389 | no KO assigned |
| cluster 2 | Asuc_1391 | no KO assigned |
| cluster 2 | Asuc_1455 | no KO assigned |
| cluster 2 | Asuc_1482 | K03319 divalent anion:Na+ symporter, DASS family |
| cluster 2 | Asuc_1542 | no KO assigned |
| cluster 2 | Asuc_1568 | K14445 solute carrier family 13 (sodium-dependent dicarboxylate transporter), member 2/3/5 |
| cluster 2 | Asuc_1578 | no KO assigned |
| cluster 2 | Asuc_1601 | K01803 triosephosphate isomerase (TIM) [EC:5.3.1.1] |
| cluster 2 | Asuc_1602 | K04561 nitric oxide reductase subunit B [EC:1.7.2.5] |
| cluster 2 | Asuc_1611 | K14055 universal stress protein E |
| cluster 2 | Asuc_1613 | K03402 transcriptional regulator of arginine metabolism |
| cluster 2 | Asuc_1655 | no KO assigned |
| cluster 2 | Asuc_1691 | K02302 uroporphyrin-III C-methyltransferase / precorrin-2 dehydrogenase / sirohydrochlorin ferrochelatase [EC:2.1.1.107 1.3.1.76 4.99.1.4] |
| cluster 2 | Asuc_1692 | K02048 sulfate transport system substrate-binding protein |
| cluster 2 | Asuc_1693 | K02046 sulfate transport system permease protein |
| cluster 2 | Asuc_1714 | K16868 tellurite methyltransferase [EC:2.1.1.265] |
| cluster 2 | Asuc_1789 | K02679 prepilin peptidase dependent protein A |
| cluster 2 | Asuc_1855 | K01708 galactarate dehydratase [EC:4.2.1.42] |
| cluster 2 | Asuc_1899 | K00965 UDPglucose--hexose-1-phosphate uridylyltransferase [EC:2.7.7.12] |
| cluster 2 | Asuc_1900 | K00849 galactokinase [EC:2.7.1.6] |
| cluster 2 | Asuc_1915 | K07821 trimethylamine-N-oxide reductase (cytochrome c), cytochrome c-type subunit TorY |
| cluster 2 | Asuc_1955 | no KO assigned |
| cluster 2 | Asuc_2049 | no KO assigned |
| cluster 2 | Asuc_2057 | K09125 uncharacterized protein |
| cluster 2 | Asuc_2101 | no KO assigned |
| cluster 2 | Asuc_2109 | K03980 putative peptidoglycan lipid II flippase |
| cluster 3 | Asuc_0014 | K02902 large subunit ribosomal protein L28 |
| cluster 3 | Asuc_0729 | K01933 phosphoribosylformylglycinamidine cyclo-ligase [EC:6.3.3.1] |
| cluster 3 | Asuc_0730 | K11175 phosphoribosylglycinamide formyltransferase 1 [EC:2.1.2.2] |
| cluster 3 | Asuc_0763 | K01491 methylenetetrahydrofolate dehydrogenase (NADP+) / methenyltetrahydrofolate cyclohydrolase [EC:1.5.1.5 3.5.4.9] |
| cluster 3 | Asuc_0894 | no KO assigned |
| cluster 3 | Asuc_0984 | no KO assigned |
| cluster 3 | Asuc_0985 | K01940 argininosuccinate synthase [EC:6.3.4.5] |
| cluster 3 | Asuc_1052 | K03210 preprotein translocase subunit YajC |
| cluster 3 | Asuc_1059 | K02348 ElaA protein |
| cluster 3 | Asuc_1147 | K00602 phosphoribosylaminoimidazolecarboxamide formyltransferase / IMP cyclohydrolase [EC:2.1.2.3 3.5.4.10] |
| cluster 3 | Asuc_1148 | K01945 phosphoribosylamine--glycine ligase [EC:6.3.4.13] |
| cluster 3 | Asuc_1173 | K02014 iron complex outermembrane recepter protein |
| cluster 3 | Asuc_1281 | K03605 hydrogenase maturation protease [EC:3.4.23.-] |
| cluster 3 | Asuc_1282 | no KO assigned |
| cluster 3 | Asuc_1283 | K04653 hydrogenase expression/formation protein HypC |
| cluster 3 | Asuc_1443 | K01716 3-hydroxyacyl-[acyl-carrier protein] dehydratase / trans-2-decenoyl-[acyl-carrier protein] isomerase [EC:4.2.1.59 5.3.3.14] |
| cluster 3 | Asuc_1598 | no KO assigned |
| cluster 3 | Asuc_1663 | K07243 high-affinity iron transporter |
| cluster 3 | Asuc_1709 | K03558 membrane protein required for colicin V production |
| cluster 3 | Asuc_1710 | K09899 uncharacterized protein |
| cluster 3 | Asuc_1750 | K02494 outer membrane lipoprotein LolB |
| cluster 3 | Asuc_1752 | K00948 ribose-phosphate pyrophosphokinase [EC:2.7.6.1] |
| cluster 3 | Asuc_1911 | K03571 rod shape-determining protein MreD |
| cluster 3 | Asuc_2010 | K03832 periplasmic protein TonB |
| cluster 3 | Asuc_2012 | K03561 biopolymer transport protein ExbB |
| cluster 3 | Asuc_2034 | K02569 cytochrome c-type protein NapC |
| cluster 4 | Asuc_0007 | no KO assigned |
| cluster 4 | Asuc_0023 | K07085 putative transport protein |
| cluster 4 | Asuc_0057 | K06147 ATP-binding cassette, subfamily B, bacterial |
| cluster 4 | Asuc_0069 | no KO assigned |
| cluster 4 | Asuc_0098 | no KO assigned |
| cluster 4 | Asuc_0099 | no KO assigned |
| cluster 4 | Asuc_0102 | no KO assigned |
| cluster 4 | Asuc_0103 | no KO assigned |
| cluster 4 | Asuc_0104 | no KO assigned |
| cluster 4 | Asuc_0105 | no KO assigned |
| cluster 4 | Asuc_0106 | no KO assigned |
| cluster 4 | Asuc_0107 | no KO assigned |
| cluster 4 | Asuc_0108 | K13017 UDP-2-acetamido-2-deoxy-ribo-hexuluronate aminotransferase [EC:2.6.1.98] |
| cluster 4 | Asuc_0109 | K13018 UDP-2-acetamido-3-amino-2,3-dideoxy-glucuronate N-acetyltransferase [EC:2.3.1.201] |
| cluster 4 | Asuc_0110 | K13016 UDP-N-acetyl-2-amino-2-deoxyglucuronate dehydrogenase [EC:1.1.1.335] |
| cluster 4 | Asuc_0111 | K02474 UDP-N-acetyl-D-galactosamine dehydrogenase [EC:1.1.1.-] |
| cluster 4 | Asuc_0160 | no KO assigned |
| cluster 4 | Asuc_0267 | no KO assigned |
| cluster 4 | Asuc_0349 | K01419 ATP-dependent HslUV protease, peptidase subunit HslV [EC:3.4.25.2] |
| cluster 4 | Asuc_0375 | K00262 glutamate dehydrogenase (NADP+) [EC:1.4.1.4] |
| cluster 4 | Asuc_0397 | no KO assigned |
| cluster 4 | Asuc_0398 | no KO assigned |
| cluster 4 | Asuc_0399 | no KO assigned |
| cluster 4 | Asuc_0400 | no KO assigned |
| cluster 4 | Asuc_0427 | K00757 uridine phosphorylase [EC:2.4.2.3] |
| cluster 4 | Asuc_0434 | K02783 PTS system, glucitol/sorbitol-specific IIC component |
| cluster 4 | Asuc_0435 | K02782 PTS system, glucitol/sorbitol-specific IIB component [EC:2.7.1.198] K02783 PTS system, glucitol/sorbitol-specific IIC component |
| cluster 4 | Asuc_0453 | no KO assigned |
| cluster 4 | Asuc_0503 | K01914 aspartate--ammonia ligase [EC:6.3.1.1] |
| cluster 4 | Asuc_0504 | K03718 Lrp/AsnC family transcriptional regulator, regulator for asnA, asnC and gidA |
| cluster 4 | Asuc_0591 | K04072 acetaldehyde dehydrogenase / alcohol dehydrogenase [EC:1.2.1.10 1.1.1.1] |
| cluster 4 | Asuc_0639 | K00763 nicotinate phosphoribosyltransferase [EC:6.3.4.21] |
| cluster 4 | Asuc_0640 | K01916 NAD+ synthase [EC:6.3.1.5] |
| cluster 4 | Asuc_0662 | K04077 chaperonin GroEL |
| cluster 4 | Asuc_0663 | K04078 chaperonin GroES |
| cluster 4 | Asuc_0703 | K03644 lipoyl synthase [EC:2.8.1.8] |
| cluster 4 | Asuc_0768 | K01338 ATP-dependent Lon protease [EC:3.4.21.53] |
| cluster 4 | Asuc_0838 | no KO assigned |
| cluster 4 | Asuc_0856 | K11931 biofilm PGA synthesis lipoprotein PgaB [EC:3.-.-.-] |
| cluster 4 | Asuc_0857 | K11936 poly-beta-1,6-N-acetyl-D-glucosamine synthase [EC:2.4.1.-] |
| cluster 4 | Asuc_0858 | no KO assigned |
| cluster 4 | Asuc_0877 | K01356 repressor LexA [EC:3.4.21.88] |
| cluster 4 | Asuc_0884 | K03631 DNA repair protein RecN (Recombination protein N) |
| cluster 4 | Asuc_0914 | K02809 PTS system, sucrose-specific IIB component [EC:2.7.1.-] K02810 PTS system, sucrose-specific IIC component |
| cluster 4 | Asuc_0925 | K00928 aspartate kinase [EC:2.7.2.4] |
| cluster 4 | Asuc_0946 | no KO assigned |
| cluster 4 | Asuc_1036 | K01433 formyltetrahydrofolate deformylase [EC:3.5.1.10] |
| cluster 4 | Asuc_1092 | K04043 molecular chaperone DnaK |
| cluster 4 | Asuc_1098 | no KO assigned |
| cluster 4 | Asuc_1161 | K00681 gamma-glutamyltranspeptidase / glutathione hydrolase [EC:2.3.2.2 3.4.19.13] |
| cluster 4 | Asuc_1205 | no KO assigned |
| cluster 4 | Asuc_1208 | no KO assigned |
| cluster 4 | Asuc_1250 | no KO assigned |
| cluster 4 | Asuc_1253 | no KO assigned |
| cluster 4 | Asuc_1304 | no KO assigned |
| cluster 4 | Asuc_1305 | no KO assigned |
| cluster 4 | Asuc_1306 | no KO assigned |
| cluster 4 | Asuc_1308 | no KO assigned |
| cluster 4 | Asuc_1325 | no KO assigned |
| cluster 4 | Asuc_1505 | no KO assigned |
| cluster 4 | Asuc_1521 | K07308 anaerobic dimethyl sulfoxide reductase subunit C (DMSO reductase anchor subunit) |
| cluster 4 | Asuc_1522 | K07307 anaerobic dimethyl sulfoxide reductase subunit B (DMSO reductase iron- sulfur subunit) |
| cluster 4 | Asuc_1559 | K02020 molybdate transport system substrate-binding protein |
| cluster 4 | Asuc_1574 | K00813 aspartate aminotransferase [EC:2.6.1.1] |
| cluster 4 | Asuc_1588 | K01414 oligopeptidase A [EC:3.4.24.70] |
| cluster 4 | Asuc_1589 | K05838 putative thioredoxin |
| cluster 4 | Asuc_1594 | no KO assigned |
| cluster 4 | Asuc_1600 | no KO assigned |
| cluster 4 | Asuc_1606 | K08978 bacterial/archaeal transporter family protein |
| cluster 4 | Asuc_1616 | no KO assigned |
| cluster 4 | Asuc_1617 | K06518 holin-like protein |
| cluster 4 | Asuc_1632 | K18924 paired small multidrug resistance pump |
| cluster 4 | Asuc_1642 | no KO assigned |
| cluster 4 | Asuc_1644 | no KO assigned |
| cluster 4 | Asuc_1645 | no KO assigned |
| cluster 4 | Asuc_1646 | no KO assigned |
| cluster 4 | Asuc_1648 | no KO assigned |
| cluster 4 | Asuc_1649 | no KO assigned |
| cluster 4 | Asuc_1650 | no KO assigned |
| cluster 4 | Asuc_1651 | K12660 2-dehydro-3-deoxy-L-rhamnonate aldolase [EC:4.1.2.53] |
| cluster 4 | Asuc_1652 | K03594 bacterioferritin [EC:1.16.3.1] |
| cluster 4 | Asuc_1697 | K03566 LysR family transcriptional regulator, glycine cleavage system transcriptional activator |
| cluster 4 | Asuc_1835 | no KO assigned |
| cluster 4 | Asuc_1836 | K07147 methionine sulfoxide reductase catalytic subunit [EC:1.8.-.-] |
| cluster 4 | Asuc_1977 | K09131 uncharacterized protein |
| cluster 4 | Asuc_1980 | K03885 NADH dehydrogenase [EC:1.6.99.3] |
| cluster 4 | Asuc_1988 | no KO assigned |
| cluster 4 | Asuc_1989 | no KO assigned |
| cluster 4 | Asuc_1990 | no KO assigned |
| cluster 4 | Asuc_1991 | K07080 uncharacterized protein |
| cluster 4 | Asuc_2014 | K03695 ATP-dependent Clp protease ATP-binding subunit ClpB |
| cluster 4 | Asuc_2102 | no KO assigned |
| cluster 4 | Asuc_2103 | no KO assigned |
| cluster 5 | Asuc_0015 | K02913 large subunit ribosomal protein L33 |
| cluster 5 | Asuc_0044 | K02863 large subunit ribosomal protein L1 |
| cluster 5 | Asuc_0045 | K02867 large subunit ribosomal protein L11 |
| cluster 5 | Asuc_0142 | K07792 anaerobic C4-dicarboxylate transporter DcuB |
| cluster 5 | Asuc_0312 | K00705 4-alpha-glucanotransferase [EC:2.4.1.25] |
| cluster 5 | Asuc_0313 | K00688 glycogen phosphorylase [EC:2.4.1.1] |
| cluster 5 | Asuc_0315 | K01176 alpha-amylase [EC:3.2.1.1] |
| cluster 5 | Asuc_0316 | K10110 maltose/maltodextrin transport system permease protein |
| cluster 5 | Asuc_0317 | K10109 maltose/maltodextrin transport system permease protein |
| cluster 5 | Asuc_0318 | K10108 maltose/maltodextrin transport system substrate-binding protein |
| cluster 5 | Asuc_0319 | K10108 maltose/maltodextrin transport system substrate-binding protein |
| cluster 5 | Asuc_0320 | K10111 multiple sugar transport system ATP-binding protein [EC:3.6.3.-] |
| cluster 5 | Asuc_0321 | no KO assigned |
| cluster 5 | Asuc_0322 | K02024 maltoporin |
| cluster 5 | Asuc_0323 | K05775 maltose operon periplasmic protein |
| cluster 5 | Asuc_0520 | K02959 small subunit ribosomal protein S16 |
| cluster 5 | Asuc_0525 | K02909 large subunit ribosomal protein L31 |
| cluster 5 | Asuc_0530 | K03585 membrane fusion protein, multidrug efflux system |
| cluster 5 | Asuc_0616 | no KO assigned |
| cluster 5 | Asuc_0647 | K05540 tRNA-dihydrouridine synthase B [EC:1.-.-.-] |
| cluster 5 | Asuc_0648 | K03557 Fis family transcriptional regulator, factor for inversion stimulation protein |
| cluster 5 | Asuc_0713 | no KO assigned |
| cluster 5 | Asuc_0721 | K02970 small subunit ribosomal protein S21 |
| cluster 5 | Asuc_0774 | K02967 small subunit ribosomal protein S2 |
| cluster 5 | Asuc_0775 | K02357 elongation factor Ts |
| cluster 5 | Asuc_0794 | K02008 cobalt/nickel transport system permease protein |
| cluster 5 | Asuc_0893 | K00088 IMP dehydrogenase [EC:1.1.1.205] |
| cluster 5 | Asuc_0896 | K01951 GMP synthase (glutamine-hydrolysing) [EC:6.3.5.2] |
| cluster 5 | Asuc_0972 | K03488 beta-glucoside operon transcriptional antiterminator |
| cluster 5 | Asuc_0973 | K01223 6-phospho-beta-glucosidase [EC:3.2.1.86] |
| cluster 5 | Asuc_0974 | no KO assigned |
| cluster 5 | Asuc_0975 | K02755 PTS system, beta-glucoside-specific IIA component [EC:2.7.1.-] K02756 PTS system, beta-glucoside-specific IIB component [EC:2.7.1.-] K02757 PTS system, beta-glucoside-specific IIC component |
| cluster 5 | Asuc_0982 | K02837 peptide chain release factor 3 |
| cluster 5 | Asuc_1057 | K07568 S-adenosylmethionine:tRNA ribosyltransferase-isomerase [EC:2.4.99.17] |
| cluster 5 | Asuc_1137 | no KO assigned |
| cluster 5 | Asuc_1185 | K09136 ribosomal protein S12 methylthiotransferase accessory factor |
| cluster 5 | Asuc_1190 | K01485 cytosine deaminase [EC:3.5.4.1] |
| cluster 5 | Asuc_1191 | K00549 5-methyltetrahydropteroyltriglutamate--homocysteine methyltransferase [EC:2.1.1.14] |
| cluster 5 | Asuc_1217 | no KO assigned |
| cluster 5 | Asuc_1295 | K01495 GTP cyclohydrolase I [EC:3.5.4.16] |
| cluster 5 | Asuc_1329 | K01868 threonyl-tRNA synthetase [EC:6.1.1.3] |
| cluster 5 | Asuc_1412 | K01056 peptidyl-tRNA hydrolase, PTH1 family [EC:3.1.1.29] |
| cluster 5 | Asuc_1413 | K06942 ribosome-binding ATPase |
| cluster 5 | Asuc_1415 | no KO assigned |
| cluster 5 | Asuc_1456 | K07259 D-alanyl-D-alanine carboxypeptidase / D-alanyl-D-alanine-endopeptidase (penicillin-binding protein 4) [EC:3.4.16.4 3.4.21.-] |
| cluster 5 | Asuc_1493 | K02996 small subunit ribosomal protein S9 |
| cluster 5 | Asuc_1494 | K02871 large subunit ribosomal protein L13 |
| cluster 5 | Asuc_1495 | K00836 diaminobutyrate-2-oxoglutarate transaminase [EC:2.6.1.76] |
| cluster 5 | Asuc_1496 | K13745 L-2,4-diaminobutyrate decarboxylase [EC:4.1.1.86] |
| cluster 5 | Asuc_1537 | K07133 uncharacterized protein |
| cluster 5 | Asuc_1540 | no KO assigned |
| cluster 5 | Asuc_1541 | no KO assigned |
| cluster 5 | Asuc_1612 | K00024 malate dehydrogenase [EC:1.1.1.37] |
| cluster 5 | Asuc_1626 | no KO assigned |
| cluster 5 | Asuc_1627 | no KO assigned |
| cluster 5 | Asuc_1707 | K03075 preprotein translocase subunit SecG |
| cluster 5 | Asuc_1743 | K11069 spermidine/putrescine transport system substrate-binding protein |
| cluster 5 | Asuc_1917 | K02518 translation initiation factor IF-1 |
| cluster 5 | Asuc_1920 | K06193 protein PhnA |
| cluster 5 | Asuc_1992 | K03580 ATP-dependent helicase HepA [EC:3.6.4.-] |
| cluster 5 | Asuc_2000 | K02078 acyl carrier protein |
| cluster 5 | Asuc_2043 | K09890 alternative ribosome-rescue factor |
| cluster 5 | Asuc_2056 | no KO assigned |
| cluster 5 | Asuc_2082 | no KO assigned |
| cluster 5 | Asuc_2117 | K02914 large subunit ribosomal protein L34 |
| cluster 6 | Asuc_0005 | K03777 D-lactate dehydrogenase [EC:1.1.1.28] |
| cluster 6 | Asuc_0051 | K03563 carbon storage regulator |
| cluster 6 | Asuc_0052 | K01872 alanyl-tRNA synthetase [EC:6.1.1.7] |
| cluster 6 | Asuc_0053 | K06149 universal stress protein A |
| cluster 6 | Asuc_0080 | no KO assigned |
| cluster 6 | Asuc_0081 | K10439 ribose transport system substrate-binding protein |
| cluster 6 | Asuc_0082 | K10441 ribose transport system ATP-binding protein [EC:3.6.3.17] |
| cluster 6 | Asuc_0083 | K10440 ribose transport system permease protein |
| cluster 6 | Asuc_0138 | K09794 uncharacterized protein |
| cluster 6 | Asuc_0140 | K01424 L-asparaginase [EC:3.5.1.1] |
| cluster 6 | Asuc_0163 | K04047 starvation-inducible DNA-binding protein |
| cluster 6 | Asuc_0171 | K00615 transketolase [EC:2.2.1.1] |
| cluster 6 | Asuc_0237 | K03476 L-ascorbate 6-phosphate lactonase [EC:3.1.1.-] |
| cluster 6 | Asuc_0366 | no KO assigned |
| cluster 6 | Asuc_0367 | no KO assigned |
| cluster 6 | Asuc_0611 | K02505 protein transport protein HofC |
| cluster 6 | Asuc_0691 | no KO assigned |
| cluster 6 | Asuc_0692 | no KO assigned |
| cluster 6 | Asuc_0748 | no KO assigned |
| cluster 6 | Asuc_0842 | K03651 3',5'-cyclic-AMP phosphodiesterase [EC:3.1.4.53] |
| cluster 6 | Asuc_1064 | K12940 aminobenzoyl-glutamate utilization protein A |
| cluster 6 | Asuc_1065 | no KO assigned |
| cluster 6 | Asuc_1073 | K03841 fructose-1,6-bisphosphatase I [EC:3.1.3.11] |
| cluster 6 | Asuc_1119 | K09748 ribosome maturation factor RimP |
| cluster 6 | Asuc_1155 | K00098 L-idonate 5-dehydrogenase [EC:1.1.1.264] |
| cluster 6 | Asuc_1362 | no KO assigned |
| cluster 6 | Asuc_1363 | K07666 two-component system, OmpR family, response regulator QseB |
| cluster 6 | Asuc_1364 | K07645 two-component system, OmpR family, sensor histidine kinase QseC [EC:2.7.13.3] |
| cluster 6 | Asuc_1441 | K05878 dihydroxyacetone kinase, N-terminal domain [EC:2.7.1.-] |
| cluster 6 | Asuc_1446 | K03704 cold shock protein (beta-ribbon, CspA family) |
| cluster 6 | Asuc_1517 | K16264 cobalt-zinc-cadmium efflux system protein |
| cluster 6 | Asuc_1528 | no KO assigned |
| cluster 6 | Asuc_1665 | no KO assigned |
| cluster 6 | Asuc_1667 | K02004 putative ABC transport system permease protein |
| cluster 6 | Asuc_1681 | K02012 iron(III) transport system substrate-binding protein |
| cluster 6 | Asuc_1682 | K02012 iron(III) transport system substrate-binding protein |
| cluster 6 | Asuc_1780 | no KO assigned |
| cluster 6 | Asuc_1788 | K02680 prepilin peptidase dependent protein B |
| cluster 6 | Asuc_1873 | K02237 competence protein ComEA |
| cluster 6 | Asuc_1897 | K10542 methyl-galactoside transport system ATP-binding protein [EC:3.6.3.17] |
| cluster 6 | Asuc_1898 | K10540 methyl-galactoside transport system substrate-binding protein |
| cluster 6 | Asuc_1970 | K03303 lactate permease |
| cluster 6 | Asuc_1971 | K18928 L-lactate dehydrogenase complex protein LldE |
| cluster 6 | Asuc_1972 | K18929 L-lactate dehydrogenase complex protein LldF |
| cluster 6 | Asuc_1973 | K00782 L-lactate dehydrogenase complex protein LldG |
