# Supplementary material for: Transcriptome analysis and anaerobic C4‐dicarboxylate transport in Actinobacillus succinogenes
Source: Microbiologyopen. 2017 Dec 12;7(3):e00565. doi: 10.1002/mbo3.565 (PMC6011838; doi:10.1002/mbo3.565)
Supplement: Supplementary file 9 [file MBO3-7-e00565-s009.docx]

**Table S5.** Number of differentially expressed genes in KEGG pathway. Pathway information of *A. succinogenes* genes were downloaded from KEGG API (<http://rest.kegg.jp/link/pathway> /asu). Genes belonging to multiple pathway have been counted more than one times.

| pathway | cluster 1 | cluster 2 | cluster 3 | cluster 4 | cluster 5 | cluster 6 | sum |
| --- | --- | --- | --- | --- | --- | --- | --- |
| asu02010 ABC transporters | 7 | 4 | 0 | 1 | 7 | 7 | 26 |
| asu03010 Ribosome | 0 | 0 | 1 | 0 | 10 | 0 | 11 |
| asu01230 Biosynthesis of amino acids | 0 | 2 | 2 | 3 | 2 | 1 | 10 |
| asu01200 Carbon metabolism | 0 | 4 | 2 | 0 | 1 | 2 | 9 |
| asu00010 Glycolysis / Gluconeogenesis | 0 | 5 | 0 | 1 | 1 | 1 | 8 |
| asu00230 Purine metabolism | 0 | 0 | 5 | 0 | 2 | 1 | 8 |
| asu00520 Amino sugar and nucleotide sugar metabolism | 1 | 2 | 0 | 4 | 0 | 0 | 7 |
| asu00920 Sulfur metabolism | 3 | 2 | 0 | 2 | 0 | 0 | 7 |
| asu00051 Fructose and mannose metabolism | 1 | 1 | 0 | 3 | 0 | 1 | 6 |
| asu00620 Pyruvate metabolism | 0 | 3 | 0 | 1 | 1 | 1 | 6 |
| asu00500 Starch and sucrose metabolism | 0 | 1 | 0 | 1 | 4 | 0 | 6 |
| asu00250 Alanine, aspartate and glutamate metabolism | 0 | 0 | 1 | 3 | 0 | 1 | 5 |
| asu02060 Phosphotransferase system (PTS) | 1 | 0 | 0 | 3 | 1 | 0 | 5 |
| asu02020 Two-component system | 0 | 0 | 0 | 0 | 1 | 3 | 4 |
| asu02030 Bacterial chemotaxis | 0 | 0 | 0 | 0 | 2 | 2 | 4 |
| asu00270 Cysteine and methionine metabolism | 0 | 0 | 0 | 2 | 2 | 0 | 4 |
| asu00260 Glycine, serine and threonine metabolism | 0 | 1 | 0 | 1 | 2 | 0 | 4 |
| asu00670 One carbon pool by folate | 0 | 0 | 3 | 1 | 0 | 0 | 4 |
| asu00020 Citrate cycle (TCA cycle) | 0 | 3 | 0 | 0 | 1 | 0 | 4 |
| asu00564 Glycerophospholipid metabolism | 4 | 0 | 0 | 0 | 0 | 0 | 4 |
| asu00030 Pentose phosphate pathway | 0 | 0 | 1 | 0 | 0 | 2 | 3 |
| asu00460 Cyanoamino acid metabolism | 0 | 0 | 0 | 2 | 0 | 1 | 3 |
| asu00053 Ascorbate and aldarate metabolism | 0 | 2 | 0 | 0 | 0 | 1 | 3 |
| asu00561 Glycerolipid metabolism | 2 | 0 | 0 | 0 | 0 | 1 | 3 |
| asu00350 Tyrosine metabolism | 0 | 1 | 0 | 2 | 0 | 0 | 3 |
| asu01210 2-Oxocarboxylic acid metabolism | 0 | 0 | 0 | 2 | 1 | 0 | 3 |
| asu00910 Nitrogen metabolism | 0 | 2 | 0 | 1 | 0 | 0 | 3 |
| asu00330 Arginine and proline metabolism | 0 | 1 | 0 | 1 | 1 | 0 | 3 |
| asu00630 Glyoxylate and dicarboxylate metabolism | 0 | 1 | 0 | 1 | 1 | 0 | 3 |
| asu00040 Pentose and glucuronate interconversions | 0 | 3 | 0 | 0 | 0 | 0 | 3 |
| asu00550 Peptidoglycan biosynthesis | 1 | 1 | 0 | 0 | 1 | 0 | 3 |
| asu00680 Methane metabolism | 0 | 0 | 0 | 0 | 1 | 1 | 2 |
| asu00970 Aminoacyl-tRNA biosynthesis | 0 | 0 | 0 | 0 | 1 | 1 | 2 |
| asu00760 Nicotinate and nicotinamide metabolism | 0 | 0 | 0 | 2 | 0 | 0 | 2 |
| asu03018 RNA degradation | 0 | 0 | 0 | 2 | 0 | 0 | 2 |
| asu00071 Fatty acid degradation | 0 | 1 | 0 | 1 | 0 | 0 | 2 |
| asu00625 Chloroalkane and chloroalkene degradation | 0 | 1 | 0 | 1 | 0 | 0 | 2 |
| asu00626 Naphthalene degradation | 0 | 1 | 0 | 1 | 0 | 0 | 2 |
| asu00860 Porphyrin and chlorophyll metabolism | 0 | 1 | 0 | 1 | 0 | 0 | 2 |
| asu01220 Degradation of aromatic compounds | 0 | 1 | 0 | 1 | 0 | 0 | 2 |
| asu00240 Pyrimidine metabolism | 0 | 0 | 0 | 1 | 1 | 0 | 2 |
| asu00052 Galactose metabolism | 0 | 2 | 0 | 0 | 0 | 0 | 2 |
| asu03060 Protein export | 0 | 0 | 1 | 0 | 1 | 0 | 2 |
| asu03070 Bacterial secretion system | 0 | 0 | 1 | 0 | 1 | 0 | 2 |
| asu00190 Oxidative phosphorylation | 0 | 0 | 0 | 1 | 0 | 0 | 1 |
| asu00300 Lysine biosynthesis | 0 | 0 | 0 | 1 | 0 | 0 | 1 |
| asu00360 Phenylalanine metabolism | 0 | 0 | 0 | 1 | 0 | 0 | 1 |
| asu00400 Phenylalanine, tyrosine and tryptophan biosynthesis | 0 | 0 | 0 | 1 | 0 | 0 | 1 |
| asu00401 Novobiocin biosynthesis | 0 | 0 | 0 | 1 | 0 | 0 | 1 |
| asu00430 Taurine and hypotaurine metabolism | 0 | 0 | 0 | 1 | 0 | 0 | 1 |
| asu00480 Glutathione metabolism | 0 | 0 | 0 | 1 | 0 | 0 | 1 |
| asu00650 Butanoate metabolism | 0 | 0 | 0 | 1 | 0 | 0 | 1 |
| asu00785 Lipoic acid metabolism | 0 | 0 | 0 | 1 | 0 | 0 | 1 |
| asu00280 Valine, leucine and isoleucine degradation | 0 | 1 | 0 | 0 | 0 | 0 | 1 |
| asu00332 Carbapenem biosynthesis | 0 | 1 | 0 | 0 | 0 | 0 | 1 |
| asu00562 Inositol phosphate metabolism | 0 | 1 | 0 | 0 | 0 | 0 | 1 |
| asu00640 Propanoate metabolism | 0 | 1 | 0 | 0 | 0 | 0 | 1 |
| asu00730 Thiamine metabolism | 0 | 1 | 0 | 0 | 0 | 0 | 1 |
| asu04122 Sulfur relay system | 0 | 1 | 0 | 0 | 0 | 0 | 1 |
| asu00450 Selenocompound metabolism | 0 | 0 | 0 | 0 | 1 | 0 | 1 |
| asu00790 Folate biosynthesis | 0 | 0 | 0 | 0 | 1 | 0 | 1 |
| asu01501 beta-Lactam resistance | 0 | 0 | 0 | 0 | 1 | 0 | 1 |
| asu00061 Fatty acid biosynthesis | 0 | 0 | 1 | 0 | 0 | 0 | 1 |
| asu01212 Fatty acid metabolism | 0 | 0 | 1 | 0 | 0 | 0 | 1 |
| asu03440 Homologous recombination | 1 | 0 | 0 | 0 | 0 | 0 | 1 |
